# Sex-Dependent Changes in miRNA Expression in the Bed Nucleus of the Stria Terminalis Following Stress

**DOI:** 10.3389/fnmol.2019.00236

**Published:** 2019-10-04

**Authors:** Maria Mavrikaki, Lorena Pantano, David Potter, Maximilian A. Rogers-Grazado, Eleni Anastasiadou, Frank J. Slack, Sami S. Amr, Kerry J. Ressler, Nikolaos P. Daskalakis, Elena Chartoff

**Affiliations:** ^1^Department of Psychiatry, Harvard Medical School, McLean Hospital, Belmont, MA, United States; ^2^Harvard Chan Bioinformatics Core, Harvard School of Public Health, Harvard University, Boston, MA, United States; ^3^Translational Genomics Core, Partners Healthcare Personalized Medicine, Cambridge, MA, United States; ^4^Department of Pathology, Beth Israel Deaconess Medical Center, Harvard Medical School, Boston, MA, United States

**Keywords:** miRNAs, stress, social isolation, small RNA sequencing, BNST, sex differences

## Abstract

Anxiety disorders disproportionately affect women compared to men, which may arise from sex differences in stress responses. MiRNAs are small non-coding RNAs known to regulate gene expression through actions on mRNAs. MiRNAs are regulated, in part, by factors such as stress and gonadal sex, and they have been implicated in the pathophysiology of multiple psychiatric disorders. Here, we assessed putative sex differences in miRNA expression in the bed nucleus of the stria terminalis (BNST) – a sexually dimorphic brain region implicated in anxiety – of adult male and female rats that had been exposed to social isolation (SI) stress throughout adolescence. To assess the translational utility of our results, we assessed if childhood trauma in humans resulted in changes in blood miRNA expression that are similar to those observed in rats. Male and female Sprague-Dawley rats underwent SI during adolescence or remained group housed (GH) and were tested for anxiety-like behavior in the elevated plus maze as adults. Small RNA sequencing was performed on tissue extracted from the BNST. Furthermore, we re-analyzed an already available small RNA sequencing data set from the Grady Trauma Project (GTP) from men and women to identify circulating miRNAs that are associated with childhood trauma exposure. Our results indicated that there were greater anxiogenic-like effects and changes in BNST miRNA expression in SI versus GH females compared to SI versus GH males. In addition, we found nine miRNAs that were regulated in both the BNST from SI compared to GH rats and in blood samples from humans exposed to childhood trauma. These studies emphasize the utility of rodent models in studying neurobiological mechanisms underlying psychiatric disorders and suggest that rodent models could be used to identify novel sex-specific pharmacotherapies for anxiety disorders.

## Introduction

Early life stressors, particularly at vulnerable developmental periods such as adolescence or childhood, increase the risk for psychiatric disorders in adulthood (Chapman et al., 2004; Gillespie et al., 2009; Khoury et al., 2010; Mehta et al., 2013). Women are twice as likely as men to be diagnosed with mood disorders (Kessler, 2003). Gender differences in the prevalence of mood disorders are first evident in adolescence (Weintraub et al., 2010), raising the possibility that there are biologically and developmentally based sex differences underlying those disorders.

Adolescent girls report much higher levels of stress associated with negative interpersonal contexts than boys (Hankin et al., 2007). Previous research demonstrated sex-specific effects of adolescent social isolation (SI) on adult stress reactivity, with adult female rats demonstrating more robust adrenal responses to acute and chronic stress compared to corresponding males (Weintraub et al., 2010). Thus, adolescent SI is a translationally relevant stressor that can induce long-term and sex-specific changes in stress reactivity and behavior.

MicroRNAs (miRNAs) are small (∼22 nucleotides; nts) non-coding RNAs that inhibit gene expression (Im and Kenny, 2012; Anastasiadou et al., 2018). Mature miRNAs are processed from genes via a cascade of events that include the enzymes DROSHA and DICER, which act sequentially to convert pri-miRNAs to mature miRNAs (Im and Kenny, 2012). A single miRNA can regulate the expression of hundreds of genes and can itself be regulated by stress and gonadal sex (Sharma and Eghbali, 2014; Pfau et al., 2016). However, there is limited research on stress-regulated miRNAs and miRNA variants that demonstrate sex-specific changes. Nonetheless, some isomiRs, which are miRNAs that vary by one or more nucleotides from the reference miRNA sequence (Tan et al., 2014), have been shown to be differentially expressed in males and females (Guo et al., 2016), but little is known about their regulation or function. As such, it is possible that both stress and sex can differentially induce the expression of both reference miRNAs and isomiRs. The nucleotide differences in isomiRs allow for a range of binding affinities to, or differential selectivity for, mRNAs, which could result in a range of effects on gene expression. IsomiRs can be quantified by next generation sequencing (NGS) (Rubio et al., 2018).

The bed nucleus of the stria terminalis (BNST), part of the extended amygdala, is one of the most complex structures in the central nervous system (Daniel and Rainnie, 2016) and is a sexually dimorphic area that expresses both estrogen and androgen receptors (Lebow and Chen, 2016). The BNST integrates information from stress and reward systems (Doura and Unterwald, 2016; Faria et al., 2016). Previous research demonstrated that activation of the anterodorsal BNST (adBNST) decreases anxiety-like behavior, whereas activation of the oval nucleus of the BNST [part of the adBNST] increases anxiety-like behavior (Kim et al., 2013). The adBNST has high expression levels of corticotropin-releasing factor (CRF) and CRF receptors (Dong et al., 2001), which is essential for stress responses (Erb and Stewart, 1999). To our knowledge, the interaction between sex and adolescent stress on miRNA expression in the adBNST is unknown. Thus, we decided to focus on adBNST as it is an understudied area involved in stress response and demonstrates a sexually dimorphic profile. The present study focused on how stressful (SI) versus non-stressful (group housed, GH) housing conditions during adolescence impacted miRNA expression in the adBNST in early adulthood.

Rodent studies are widely used to assess molecular mechanisms underlying neuropsychiatric disorders; however, there is a need to develop better validated and more useful animal models (Nestler and Hyman, 2010). To achieve this goal, it is essential to compare rodent findings to human data in order to assess their translational utility. In this study, we assessed miRNA expression in the adBNST following SI in rats and we aimed to compare those data to a human stress-related already existing miRNA data set. In addition to the brain and other organs, miRNAs can also be detected in circulating biological fluids such as blood (i.e., circulating miRNAs). Under healthy conditions, miRNA expression profiles remain stable in circulating biological fluids, but can significantly change in response to pathological conditions such as prolonged or severe stress (Chen et al., 2008; Dwivedi, 2014; Grasso et al., 2014). Thus, recent research suggests that miRNAs can not only be used as therapeutic targets, but also as non-invasive diagnostic and predictive biomarkers, which is highly impactful given the ethical and practical limitations in obtaining human brain tissue (Glinge et al., 2017). Given the limitations of research using human brain tissues, it is not surprising that miRNA expression profile in the BNST has not been assessed following SI stress. Although stressful events are not always traumatic, traumatic experiences are always stressful (Willard et al., 2016). Previous research from our group assessed miRNA expression in whole blood in individuals that experienced childhood trauma (Wingo et al., 2015). Blood gene expression may not necessarily map to parallel alterations in the brain itself, although our team findings from animal models of post-traumatic stress disorder (PTSD) have shown convergence between brain and blood gene expression signatures associated with individual differences in the behavioral response to stress at the level of upstream regulators and pathways (Daskalakis et al., 2014; Lori et al., 2018). Here, we re-analyzed a previously published small RNA sequencing data set from men and women (Wingo et al., 2015) to identify circulating miRNAs associated with childhood trauma exposure that overlap with adBNST miRNAs associated with adolescent SI.

In this study, we hypothesized that adolescent SI would produce a more robust anxiogenic phenotype compared to GH conditions in adult females than in males, and increased anxiety would be accompanied by sex-specific changes in miRNA expression in the adBNST. We also hypothesized that childhood trauma experience in humans would induce changes in blood miRNA expression that are similar to those observed in the anxiety-related adBNST in rats (Daskalakis et al., 2018). These studies are important because they probe novel molecular consequences of adolescent social conditions that might lead to improved understanding and development of sex-specific treatments for stress-related disorders.

## Materials and Methods

### Animals

Timed-pregnant female Sprague-Dawley rats (gestation day 15) were purchased from Charles River Laboratories (Wilmington, MA, United States) based on previously published work (Brenhouse et al., 2009; Holland et al., 2014). Once the pregnant dams arrived, they had an additional 7 days to habituate to the animal colony before giving birth. Although we appreciate that shipment of the pregnant dams can be a stressor, no differences in average litter size (9–12 pups), sex ratio, or weight/health of the pups were noticed in the offspring. After birth, male and female pups were weaned on postnatal day 21 (PD 21) and housed either singly (socially isolated; SI) or in groups of three same-sex littermates (GH; controls). Males and females were housed separately in the same rat colony room, which is on a 12-h light–dark cycle (lights on 7:00 am). All experiments were conducted during the light phase. Rats were treated according to the guidelines recommended by the Animal Care and Use Committee of McLean Hospital and by the National Institutes of Health guide for the care and use of Laboratory animals.

### Elevated Plus Maze

After weaning (PD 21), SI and GH rats were maintained under these housing conditions for 6 weeks, as in Butler et al. (2014) and were tested for anxiety-like behavior using the elevated plus maze (EPM) when they reached adulthood (9 weeks old). The EPM was performed as in Knoll et al. (2007). Rats were transported to a holding room with dim white light (LED A19 lamp, 40 W) 1 h before testing. The maze was elevated 85 cm from the ground and consisted of two opposing open arms and two opposing closed arms (40 cm wall height). Rats were placed into the maze facing an open arm (for consistency) and the 5-min sessions were digitally captured by a camera (EverFocus Polestar II, EQ 610) mounted to the ceiling. Digital recordings were analyzed by a viewer blinded to treatment conditions for time spent in open and closed arms, as well as number of entries into open and closed arms. The maze was cleaned thoroughly with 70% ethanol between rats to avoid odors influencing behavior – particularly between males and females. The experiment was performed under red light (between 12:00 amd 3:30 pm) and in the presence of low volume white noise (60 dB). After testing, rats remained in the holding room (range: 1–4 h) until the last rat was tested. The testing order of the rats was randomized by treatment group so that each treatment group contained rats that remained in the holding room for times that spanned the 1–4 h. Rats were then transferred to a different procedure room a few minutes before they were killed for tissue extraction.

### Tissue Collection and RNA Extraction

Rats were exposed to CO_2_ for 10 s prior to rapid decapitation 1–4 h after completion of the EPM test (time of death was between 3:00 and 5:00 pm). Trunk blood was collected in 15 ml BD Vacutainer PPT plasma preparation tube (BD Biosciences) to assess estradiol levels in female rats (see the Supplementary Material) and brains were snap-frozen in isopentane on dry-ice and then stored at −80°C until use. The adBNST was punched from frozen brains as described in Chartoff et al. (2016). Briefly, frozen brains were coronally sectioned on a cryostat (HM 505 E; Microm, Walldorf, Germany) until the anterior BNST was exposed (Bregma 0.00 mm), based on the atlas of Paxinos and Watson (2007). The anterior commissure (ac) and the ventral extension of the lateral ventricle were used as anatomical hallmarks to center the corer between the ac and the ventricle. The corer was right against the boundary of the emerging globus pallidus (GP). Bilateral tissue punches 0.5–0.75 mm in length were taken with a 1-mm internal diameter corer and 1.8-mm outer diameter (Fine Science Tools, Foster City, CA, United States; Figures 2A,B; note that the punch looks bigger due to the outer diameter of the corer) and tissue was placed in Eppendorf tubes kept on dry ice and then stored at −80°C. Total RNA was extracted using Trizol reagent (Ambion, Life Technologies) and was treated with a DNA-free kit (Ambion, Life Technologies) to remove genomic DNA contamination according to Mavrikaki et al. (2016). RNA samples were split in two, and half of the samples was processed for small RNA sequencing and the other half was kept at −80°C for the validation of the sequencing using quantitative real-time polymerase chain reaction (qRT-PCR). For the small RNA sequencing female samples, we selected five samples per group that had the highest RNA integrity numbers (RIN) and that represented a range of estradiol levels (high–medium–low levels compared to the mean of the group) to correct for the estrus cycle stage. With these constraints, an *N* of five samples per group was too small to conduct reliable correlational analyses between estradiol levels and miRNA expression.

### Small RNA Sequencing

MicroRNA Libraries were prepared using the Bioo Scientific NEXTflex Small RNA-Seq kit v3 per the manufacturer’s instructions. Total RNA (215 ng) was used as input and NEXTflex adapters were ligated to the 3′- and 5′-ends of the RNA. After adapter ligations and cleanups, the adapter-ligated RNA entered reverse transcription, resulting in a cDNA first-strand synthesis product. The reverse transcription products were then isolated and amplified by PCR (22 cycles). Successful library production and isolation was confirmed by the Agilent High-Sensitivity Screentape assay. Quantification of libraries was performed using Qubit Picogreen and KAPA qRT-PCR assays. Libraries were normalized, pooled, denatured, and sequenced on the Illumina HiSeq 2500 Rapid v2 platform to generate >10 million (range 11–19 million) 50 bp single end reads per sample.

All samples were processed using the small RNA-seq pipeline implemented in the bcbio-nextgen project^1^. Raw reads were examined for quality issues using FastQC^2^ to ensure library generation and sequencing are suitable for further analysis. Adapter sequences, other contaminant sequences such as polyA tails and low-quality sequences with PHRED quality scores <5 were trimmed from reads using cutadapt (Martin, 2011). Trimmed reads were aligned to miRBase v21 (Kozomara and Griffiths-Jones, 2014) to the specific species with seqbuster (Pantano et al., 2010). As well, they were aligned to *Rattus norvegicus* genome (version rn6) using STAR (Dobin et al., 2013). The aligned genomes were used with seqcluster (Pantano et al., 2011) to characterize the whole small RNA transcriptome and classify reads into rRNA, miRNA, repeats, genes, tRNAs, and others from USCC annotation (Mangan et al., 2014). Finally, aligned reads were used with miRDeep2 (Friedlander et al., 2012), an algorithm that assesses the fit of sequenced RNAs to a biological model of miRNA generation and correct folding. Alignments were checked for evenness of coverage, rRNA content, genomic context of alignments (for example, alignments in known transcripts and introns), complexity, and other quality checks using a combination of FastQC, MultiQC (Ewels et al., 2016), and custom code inside bcbio-nextgen pipeline. Data were loaded into R with bcbioSmallRna R package^3^ and isomiRs BioC package (Pantano et al., 2016).

### Quantitative Real-Time PCR

A total of 50 ng RNA from the same line of samples used for the small RNA sequencing study was used for miRNA-specific reverse transcription using the miScript II RT kit (Qiagen) according to the manufacturer’s instructions. qRT-PCR was performed in a 384-well plate using miScript SYBR Green PCR kits (Qiagen) and miScript primer assays (Qiagen) for miR-34c-5p (MS00000238), miR-760-3p (MS00033628), miR-770-3p (MS00028539), miR-140-5p (MS00000406), miR-23b-3p (MS00033341), and RNU6B (MS00033740) using an Applied Biosystems vii7 Real-Time PCR Instrument as previously described (Mavrikaki et al., 2019). miR-23b-3p was compared to RNU6B and was used as a reference gene (Supplementary Figure 4). Expression data were analyzed according to the 2^–ΔΔCt^ method (Schmittgen and Livak, 2008).

### Comparison of Rat miRNA Data With miRNA Expression Profile in Human Participants in the Grady Trauma Project

To assess the translational utility of our results, we compared differentially expressed miRNAs from the adBNST of SI or GH male and female rats to differentially expressed miRNAs from whole blood taken from humans who had experienced childhood trauma (Wingo et al., 2015). Not all the individuals reporting high levels of trauma exposure in GTP were endorsed high symptoms of or diagnosed for PTSD or depression (Supplementary Table 7). To determine the overlap between the rat and the human miRNA lists, we used *p* < 0.05 rather than *p*adj. < 0.05 (as used in the rat study) as a cutoff for differentially expressed rat miRNAs to enhance our ability to identify changes that overlap with human data.

For the human miRNA data, we re-analyzed a previously published small RNA sequencing study (Gene Expression Omnibus GSE74162) (Wingo et al., 2015) from the Grady Trauma Project (GTP) that assessed childhood trauma using the Childhood Trauma Questionnaire (CTQ) (Bernstein et al., 2003). In that study, whole blood RNA samples from 23 subjects who participated in the GTP study were analyzed for miRNA sequencing using Illumina HiSeq 1000 (Wingo et al., 2015). For downstream analyses we kept 1117 miRNAs expressed (>0 counts) in >25% of the samples. We kept 21 samples for which we had covariate information.

After log-transformation and quantile normalization, we performed a bioinformatic analysis using the limma R-package to assess the effects of childhood trauma on miRNA expression. Childhood trauma was assessed using the total score of CTQ (this score takes into account all five subscales: emotional abuse, physical abuse, sexual abuse, emotional neglect, and physical neglect). We covaried this analysis for confounding effects of adult trauma. We analyzed data from 7 men and 14 women together due to the insufficient sample size. However, we set sex as a covariate in our analysis. In our analysis we also took into account RIN, age, and the type of the four most frequent immune cell types in each sample estimated by the CIBERSORT method (Newman et al., 2015) using RNA expression data from the same individuals (GSE67663; Illumina Human WG-6 v3.0 expression beadchip). To begin to explore if the miRNAs we detected to be associated with childhood trauma are influenced by sex hormones, we used menstrual status as a covariate in our analysis for females.

### Statistical Analysis

For the small RNA sequencing study, differential expression (DE) at the gene level was called with DESeq2 (Love et al., 2014) and *p*-adjusted value was used as a threshold for significant differences. Pathway analysis was performed using DIANA miRPath v.3 (Vlachos et al., 2015). The criteria used were: intersection of at least three miRNAs, *p*-value threshold 0.05, and MicroT threshold 0.8. For the EPM and qRT-PCR studies, statistical analysis was performed using GraphPad Prism 8. Unpaired *t*-test was used for comparisons of housing conditions per sex for EPM and two-way ANOVA (housing condition × miRNA) with repeated measures was used on miRNA for qRT-PCR. *Post hoc* analysis was performed using Fisher’s LSD. For human miRNA DE analysis using the limma R-package, we report the DE for the 188 miRNAs that were expressed in both human blood and rat BNST.

## Results

### Housing Condition During Adolescence Has Sex-Dependent Effects on Anxiety-Like Behavior in Adulthood

**FIGURE 1:**
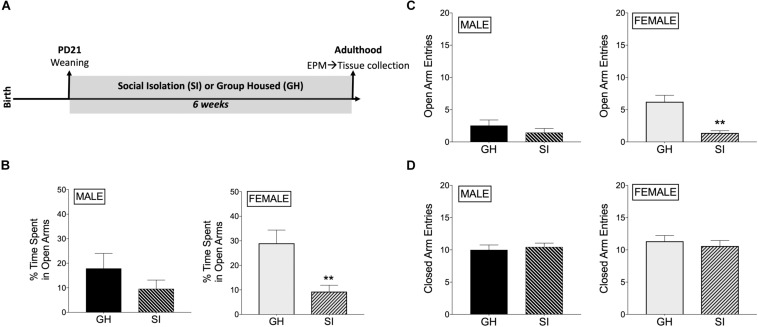
Effects of housing condition during adolescence on anxiety-like behavior in adulthood as measured in the elevated plus maze (EPM). **(A)** Experimental design of the study. **(B)** Male SI and GH rats did not differ in % time spent in open arms (TSOA) [TSOA/(TSOA + closed arm time)], whereas the % TSOA was significantly decreased in SI compared to GH female rats. **(C)** Male SI and GH rats did not differ in the number of open arm entries (OAE), whereas the number of OAE was significantly decreased in SI compared to GH female rats. **(D)** Neither male nor female SI and GH rats differed in the number of closed arm entries. *n* = 9–11/group; GH, group housed; SI, socially isolated; Asterisks indicate significant difference compared to GH control; ^∗∗^*p* < 0.01.

Rats underwent adolescent SI (or were GH) for 6 weeks and were then tested for anxiety-like behavior in the EPM when they reached adulthood (Figure 1A). SI females had decreased % time spent in the open arms of the EPM compared to GH females [*t*(17) = 3.39, *p* < 0.005; Figure 1B], whereas this difference in males was not significant [*t*(18) = 1.23, *p* = *0.23*; Figure 1B]. Similarly, SI females, but not males, had fewer open arm entries compared to GH rats [females: *t*(17) = 4.72, *p* < 0.0005; males: *t*(18) = 1.07, *p* = 0.3; Figure 1C], which taken together suggests a greater anxiogenic effect of SI in females compared to males. Finally, both SI females and males had similar closed arm entries [females: *t*(17) = 0.59, *p* = 0.5; males: *t*(18) = 0.47, *p* = 0.6; Figure 1D], suggesting that there was not a general alteration in locomotor activity due to social stress or sex. Of note, GH females tended to spend more time in the open arms than GH males [*t*(16) = 1.618, *p* = 0.12; comparing GH data from Figure 1B, male and Figure 1B, female], whereas SI females and SI males spent similar time in the open arms [t(19) = 0.094, p = 0.9; comparing SI data from Figure 1B, male and Figure 1B, female]. SI did not significantly affect estradiol levels in adulthood [*t*(17) = 1.047, *p* = 0.3; Supplementary Figure 5].

### Housing Condition During Adolescence Has Sex-Dependent Effects on miRNA Expression in the adBNST in Adulthood

**FIGURE 2:**
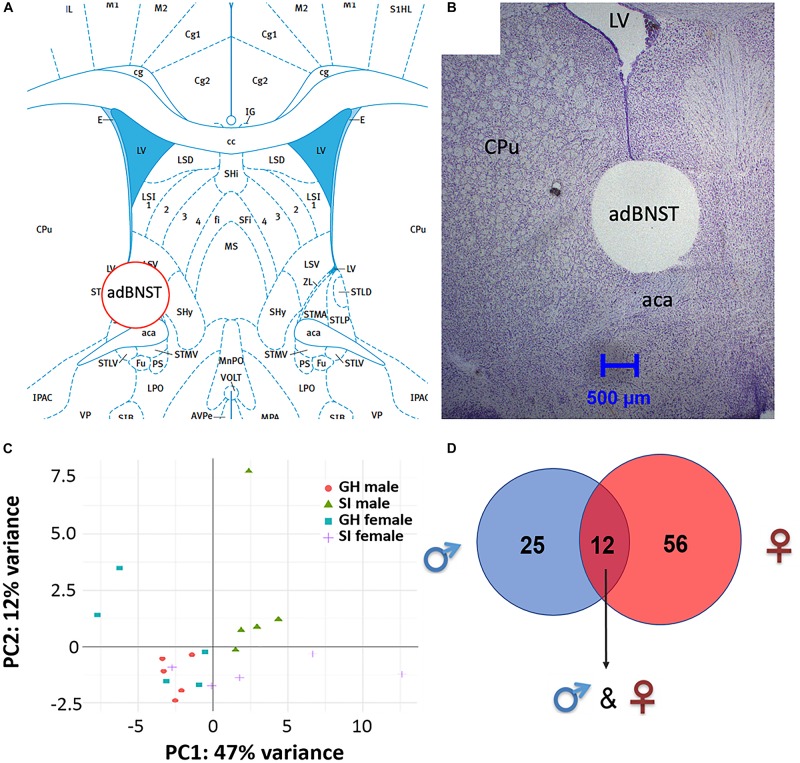
Visualization of the extracted anterodorsal bed nucleus of the stria terminalis (adBNST) and overview of differentially expressed (DE) miRNAs from the adBNST measured using small RNA sequencing. **(A)** Representative coronal brain atlas plate (Paxinos and Watson, 2007) of brain punch location. **(B)** Cresyl violet stained tissue section showing verification of brain punch location of rat adBNST. Note that the internal diameter of the corer used is 1-mm, which is approximately the width and height of the adBNST. Importantly, the outer diameter of the corer is 1.8-mm, which means that the cresyl violet histology pictures show a “hole” that is larger than the actual tissue punch. We processed tissue sections for each brain to examine the accuracy of our BNST punches (both beginning and end of tissue punch), and we discard data from any brains that have off-target BNST punches. Note that for this study, all BNST punches were on-target. **(C)** Principal component analysis showing the first two components of the regularized log2 transformed miRNA abundance data. Every dot represents a sample and they are colored by the condition information (see the legend). **(D)** A Venn diagram that indicates the number of DE miRNAs that reached significance in SI compared to GH controls in the adBNST in males (blue), females (red), or in both sexes (overlap of blue and red). GH, group housed; SI, socially isolated.

Bioinformatic analysis of the small RNA sequencing results from the adBNST (Figures 2A,B) indicated that approximately 80% of the reads corresponded to miRNAs. Principal component analysis (PCA) of all miRNAs measured indicated that most of the samples cluster together and the largest variability is observed in the female samples (Figure 2C).

**FIGURE 3:**
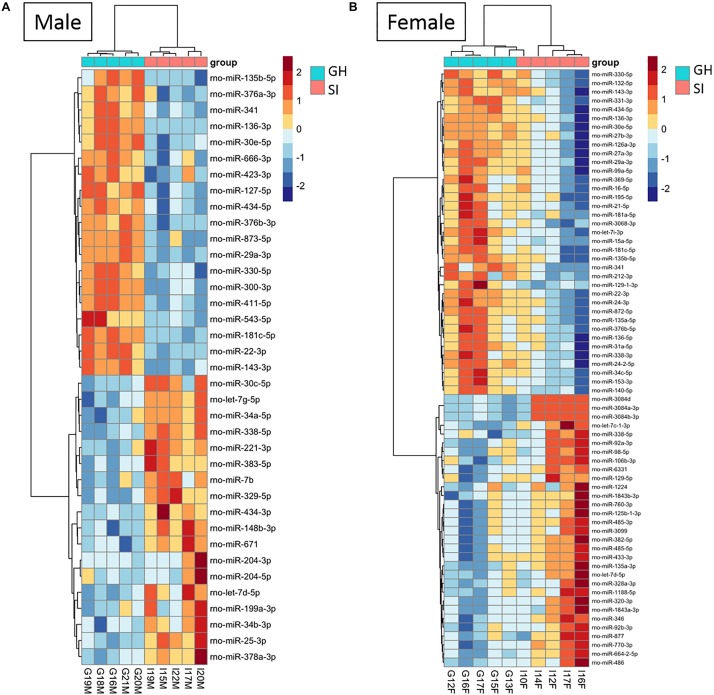
Housing condition during adolescence induces sex-specific miRNA expression in the anterodorsal bed nucleus of the stria terminalis (adBNST) in adulthood. Color represents the miRNA *z*-score expression. The median of the expression is used for each group. DE miRNAs between SI and GH in **(A)** males and **(B)** females; *n* = 5/group; GH, group housed; SI, socially isolated.

To compare the miRNA expression patterns between male GH vs. SI and female GH vs. SI, we performed a DE analysis. We used DESeq2 at the gene level and set the threshold for significant differences to *p*-adjusted (*p*adj.) < 0.05. As adBNST is a sexually dimorphic area, we first compared miRNA expression between male and female GH control rats. We found that only rno-miR-3084a/b/d was differentially expressed between male and female GH controls. Thus, we proceeded with the analysis that compared male GH vs. SI and female GH vs. SI. This analysis revealed that a total of 37 miRNAs were differentially expressed in the male, 68 miRNAs were differentially expressed in the female, and 12 miRNAs overlapped and were differentially expressed in both male and female adBNST of SI compared to GH rats (*p*adj. < 0.05; Figures 2D, 3A,B and Supplementary Table 1). The majority of those miRNAs were downregulated (10 miRNAs), and two were upregulated in the SI compared to GH condition in both sexes. From the male-specific miRNAs that were differentially expressed in SI compared to GH rats, the majority of those miRNAs (16 miRNAs) were upregulated but others (9 miRNAs) were downregulated (Supplementary Table 2). From the female-specific miRNAs that were differentially expressed in SI compared to GH rats, 29 were upregulated and 27 were downregulated (Supplementary Table 3). Note that for the miRNA analysis, all isomiRs for each reference miRNA are merged. Our sequencing analysis also identified one novel DE miRNA in both males and females, eight novel DE miRNAs in males only, and five novel DE miRNAs in females only (Supplementary Table 4).

Seqbuster of small RNA Seq reads were aligned to rat miRBase v21, which allowed for aligning of sequences to chromosomes. Our results indicate that most of the miRNA precursors that were DE between SI and GH conditions map onto Chromosome 6. Indeed, we found that a cluster on Chromosome 6 in males included rno-miR-329, rno-miR-411, rno-miR-543, and rno-miR-666, and a cluster in females included rno-miR-369, rno-miR-382, and rno-miR-485. Of the differentially expressed (SI vs. GH) miRNAs identified in this study, we found only one miRNA gene in females (miR-98) and one in males (miR-221) that map onto Chromosome X, and none onto Chromosome Y (Figure 6).

### Pathway Analysis Reveals Sex-Specific Pathway Activation in adBNST Tissue From SI Compared to GH Rats

**FIGURE 4:**
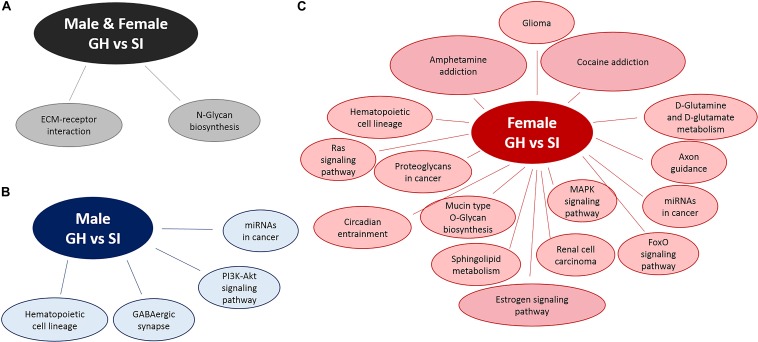
Pathway analysis of DE miRNAs using DIANA miRPath v.3. Pathway analysis for DE miRNAs between SI and GH conditions in **(A)** both males and females, **(B)** males only, and **(C)** females only. GH, group housed; SI, socially isolated.

We used DIANA miRPath v.3 to identify pathways in which DE miRNAs might be involved (Figure 4). Based on an analysis, that assesses pathways involved based on mRNA targets, using an intersection of three miRNAs and MicroT Threshold 0.8, we identified two such pathways from differentially expressed miRNAs from both males and females: ECM–receptor interaction and *N*-glycan biosynthesis (Figure 4A). Similar analysis revealed four pathways from DE, male-specific miRNAs: hematopoietic cell lineage, GABAergic synapse, microRNAs in cancer, and the PI3K-Akt signaling pathway (Figure 4B). Pathway analysis for the DE female-specific miRNAs revealed a total of 16 pathways, including estrogen signaling, cocaine and amphetamine addiction pathways, MAPK signaling, circadian entrainment, axon guidance, and D-glutamine and D-glutamate metabolism (Figure 4C).

### Validation of miRNA Sequencing Results

**FIGURE 5:**
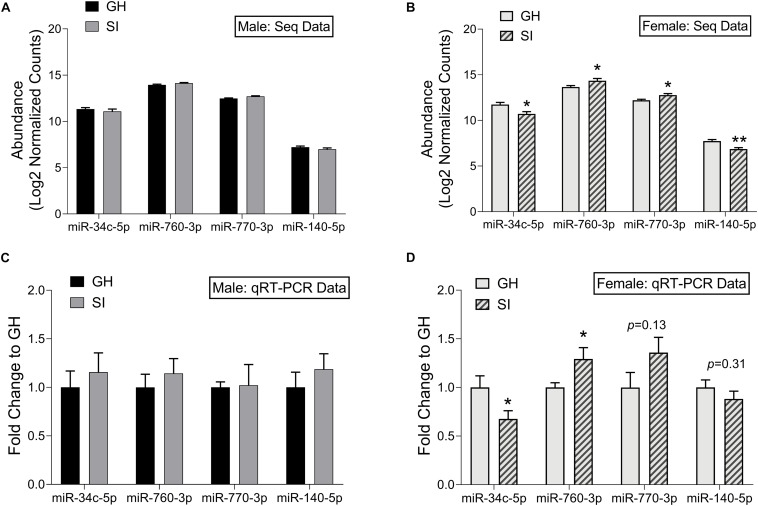
Validation of small RNA sequencing results using qRT-PCR. Log2 normalized counts (abundance) of miRNAs in SI and GH conditions as measured by small RNA sequencing; males **(A)** and females **(B)**. *n* = 5 rats/group. Fold induction (to GH condition) of qRT-PCR results in the adBNST for levels of miRNAs shown in **(A,B)**; Males **(C)** and females **(D)**. *n* = 6–9 rats/group. ^∗^*p* < 0.05, ^∗∗^*p* < 0.01, *p* = *X* (trend for significance), compared to GH condition for each miRNA. GH, group housed; SI, socially isolated.

To confirm our small RNA sequencing results, we chose representative, DE miRNAs, and performed qRT-PCR on the same RNA samples that we used in the sequencing study as well as on an additional set of samples from rats tested in the EPM but not in the sequencing study due to space on the sequencing platform. We chose to validate select miRNAs that were DE in females. These were either among the top 10 significantly regulated miRNAs (miR-760-3p and miR-770-3p), or were found within pathways of interest including MAPK signaling and addiction pathways (miR-34c-5p and miR-140-5p). We first identified a miRNA, miR-23b-3p, that was stably expressed across groups in the adBNST in our sequencing study (mean expression 14.03; *SD* 0.098) and confirmed stable expression using qRT-PCR (*p* > 0.05; Supplementary Figure 4). Therefore, we proceeded to use miR-23b-3p for normalization across samples. Of the four miRNAs we chose to validate, none were significantly different between SI or GH males in either the sequencing results (Figure 5A) or in the qRT-PCR results (Figure 5C). In females, all four miRNAs were significantly regulated by housing condition in the sequencing study (housing × miRNA interaction: *F*_(__3__,__24__)_ = 11.44, *p* < 0.001; Figure 5B). In the female qRT-PCR validation, miRNA expression depended on an interaction between housing and miRNA [*F*_(__3__,__39__)_ = 4.30, *p* < 0.05; Figure 5D], with *post hoc* analysis showing significant differences between SI and GH conditions for miR-34c-5p and miR-760-3p and trends for differences for miR-770-3p and miR-140-5p. In all cases, the direction of regulation matched that of the sequencing results.

### Housing Condition During Adolescence Has Sex-Dependent Effects on isomiR Expression in the adBNST in Adulthood

One of the main advantages of using RNA sequencing is its ability to detect changes at a single nucleotide level and detect different miRNA isomers (isomiRs); these include both the reference sequences and also sequences with 1–2 nt variations in the 3p- or 5p-end of the reference miRNA sequence. Using DE analysis, we found a total of 15 isomiRs that were differentially expressed in SI compared to GH males only, 120 isomiRs that were differentially expressed in SI compared to GH females only and five isomiRs that were differentially expressed in SI compared to GH rats of both sexes (Supplementary Figures 1A,B,2,3 for the PCA analysis of all isomiRs measured). Interestingly, in males we identified only seven isomiRs that do not correspond to the reference miRNA sequences (Supplementary Table 5). In females, there were 60 isomiRs that did not correspond to reference miRNA sequences and were significantly different between GH and SI (Supplementary Table 6). Note that we did not observe any significant differences in isomiR expression when we compared the GH males to the GH females.

### Housing Condition During Adolescence in Rats and Childhood Trauma in Humans Induce Similar Changes in miRNAs

Our bioinformatic analysis revealed 15 miRNAs in human blood that were differentially regulated following childhood trauma that are also expressed in the rat adBNST. Of those, nine miRNAs were DE between SI and GH conditions in the rats (Table 1). Focusing on miRNAs regulated specifically by childhood trauma in the human samples, we found five miRNAs (miR-106b-3p, miR-181a-5p, miR-125b-1-3p, miR-423-5p, and let-7e-5p) that are DE in SI compared to GH female, and two miRNAs (miR-204-5p and let-7i-5p) DE in SI compared to GH male, and two miRNAs (miR-146a-5p and let-7b-3p) that are DE in SI compared to GH male and female, rat adBNST (Table 1). Our analysis showed that the menstrual status of the females did not affect the DE miRNAs.

**TABLE 1:** miRNAs regulated by housing condition in rats and by trauma in humans.

**miRNA ID**	**Sex specificity in the rat study**	**Human study**
miR-204-5p	Male only	Childhood trauma
miR-106b-3p	Female only	Childhood trauma
miR-181a-5p	Female only	Childhood trauma
miR-125b-1-3p	Female only	Childhood trauma
miR-423-5p	Female only	Childhood trauma
let-7e-5p	Female only	Childhood trauma
let-7i-5p	Male only	Childhood trauma
miR-146a-5p	Both sexes	Childhood trauma
let-7b-3p	Both sexes	Childhood trauma

*Overlapping miRNAs regulated by housing condition in the rat anterodorsal bed nucleus of the stria terminalis (adBNST) and by childhood trauma in human blood samples.*

## Discussion

This study demonstrates that social housing conditions during adolescence influence anxiety-like behavior in adulthood and produce sex-dependent miRNA signatures in the adBNST, a sexually dimorphic brain region integral to regulating anxiety (Kim et al., 2013; Doura and Unterwald, 2016; Lebow and Chen, 2016). Specifically, females that underwent SI during adolescence showed increased anxiety-like behavior as adults in the EPM compared to GH females, whereas there was no significant effect of adolescent SI on EPM behavior in males. Our small RNA sequencing study and the relevant bioinformatic analysis indicated that the majority of DE miRNAs in the adBNST between SI and GH conditions were sex-specific. In addition, pathway analysis demonstrated that the DE miRNAs are involved in different signaling pathways in males and females. For example, we found that in the female adBNST, miRNAs that are differentially expressed between SI and GH conditions are involved in cocaine and amphetamine addiction pathways as well as in estrogen and MAPK intracellular pathways. However, we found that only one miRNA (rno-miR-3084a/b/d) was differentially expressed between male and female GH controls. By comparing our rat miRNA expression profile based on the different housing conditions with already existing human miRNA data obtained from blood of individuals who experienced trauma (participants in the GTP), we identified nine miRNAs (that are conserved between humans and rats), as stress- and trauma-regulated miRNAs in rats and humans. This finding supports the utility of rodent studies in the effort to better characterize mechanisms underlying human psychopathology.

Adolescent SI has been shown to induce long-lasting effects on stress reactivity and increase anxiety-like behavior in adulthood, an effect that may be sex-specific (McCormick et al., 2004; Weintraub et al., 2010; Skelly et al., 2015). The current study shows that SI compared to GH induced a robust anxiogenic phenotype in female rats, but this effect did not reach significance in male rats. This is supported in the current study by the decreased % time spent in, and entries into, the open arms in females that underwent SI compared to GH conditions. Although we observed similar trends in the males, the effects were not significant. Indeed, our data suggest that it may be GH conditions that are more anxiogenic in males compared to females: quantitatively, GH females spent a greater % of time in the open arms than GH males, an effect that did not reach statistical significance. An early study showed that group housing and overcrowded cages produced a strong stress response in male rats but “calmed” females (Brown and Grunberg, 1995). This effect might be observed in the males due to the social hierarchy, that others have shown that can be anxiogenic in male rodents (Horii et al., 2017). Previous studies using a similar SI paradigm with adolescent onset in male Sprague-Dawley rats assessed anxiety-like behavior using an EPM and showed that control male rats demonstrate ∼40 s (13%) time spent in open arms (Bledsoe et al., 2011), percent lower than what we observed in the present study (18%). Our results are consistent with previous research showing that adult female rats that underwent adolescent SI demonstrated increased adrenal responses to acute or repeated stress (Weintraub et al., 2010). However, other studies using Long-Evans rats showed that adolescent SI is anxiogenic in male rats (Chappell et al., 2013).

Consistent with the more robust anxiogenic phenotype observed in the SI female rats, we observed greater DE in miRNA expression in the female, compared to the male adBNST. Previous research has shown that adBNST is involved in anxiety-like behavior (Kim et al., 2013; Doura and Unterwald, 2016), which raises the possibility that the observed changes in miRNAs might promote anxiety-like behavior. There was little overlap of miRNA changes between males and females. A previous study by Pfau et al. (2016) using subchronic variable stress followed by small RNA sequencing in the nucleus accumbens (NAc) indicated little overlap of miRNA changes between males and females. In addition, this study also revealed that some miRNAs were up-regulated whereas others were down-regulated following stress (Pfau et al., 2016). Taken together, these findings suggest that males and females process stress differently.

Pathway analysis is a first-choice option to obtain better insight into the underlying biology of differentially expressed genes as it reduces complexity but has increased explanatory power (Khatri et al., 2012). MiRNA-related pathway analysis typically assesses mRNA target predictions (in some cases experimentally proven) followed by a targeted pathway analysis. In this study, we used DIANA miRPath v.3. (Vlachos et al., 2015) to assess pathways in which differentially expressed miRNAs can be involved. DIANA miRPath v.3 assesses miRNA–mRNA target interactions and has incorporated more than 600,000 experimentally supported miRNA targets (Vlachos et al., 2015). As multiple miRNAs have been shown to co-act to regulate molecular pathways, one of the advantages of DIANA miRPath v.3 is that it allows the identification of genes and pathways that are targeted by multiple miRNAs (Vlachos et al., 2015) (in our case minimum of three miRNAs regulating the same target gene). Thus, this type of pathway analysis can provide more biologically relevant results. In females, but not males, we found that DE miRNAs between SI and GH groups were implicated in addiction pathways. This result is intriguing, since SI has been shown to increase drug intake (Bozarth et al., 1989; Raz and Berger, 2010), and it has previously been suggested that miRNAs regulate interactions between stress and drug addiction (Doura and Unterwald, 2016). This finding is also consistent with human literature suggesting there is more of an association of childhood maltreatment and drug dependence in women compared to men (Enoch, 2011). In addition, our results indicate that DE miRNAs between SI and GH conditions in females only are involved in multiple other pathways, including the estrogen signaling pathway, the mitogen-activated protein kinase (MAPK) signaling pathway, the circadian entrainment pathway, and the axon guidance pathway. Further studies that assess the effects of these miRNAs on their relevant mRNA targets are required to confirm their involvement on the aforementioned pathways.

Quantitative real-time polymerase chain reaction (qRT-PCR) is a powerful tool to assess miRNA changes under different experimental conditions, but reliable results depend upon proper normalization to suitable reference genes (Mase et al., 2017). Although RNU6B is typically used for normalization of miRNA expression (Schwarzenbach et al., 2015), it is a small nucleolar RNA rather than a miRNA. Therefore, it lacks the biochemical characteristics of miRNAs (Schwarzenbach et al., 2015). In the current study, our small RNA sequencing results showed that miRNA miR-23b-3p is stably expressed in the adBNST, a finding supported by the small standard deviation (*SD* = 0.09) in expression among all samples. To validate the sequencing results and the utility of miR-23b-3p as a reference gene in the adBNST, we measured the expression of miR-23b-3p and RNU6B using qRT-PCR and confirmed that miR-23b-3p is stably expressed, thus providing a more biologically relevant approach to normalize miRNA expression in our adBNST samples (Supplementary Figure 4). In addition, using qRT-PCR we validated some of the miRNA sequencing results (miR-34c-5p, miR-760-3p, miR-770-3p, and miR-140-5p) and found similar changes to the small RNA sequencing study. These results support the reliability of results obtained using sequencing.

**FIGURE 6:**
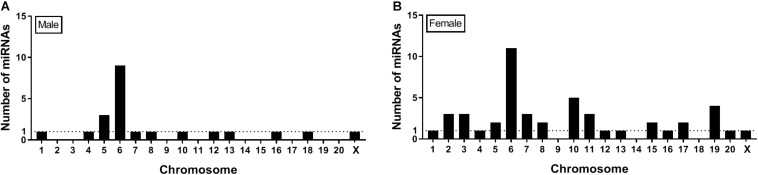
Location on chromosomes of miRNA precursors that are differentially expressed between SI and GH. Seqbuster of small RNA Seq reads were aligned to rat miRBase v21, which allowed for aligning of sequences to chromosomes. Most of the miRNA precursors that are differentially expressed between SI and GH are located on chromosome 6. **(A)** miRNA precursors that are differentially expressed between SI and GH in males only. **(B)** miRNA precursors that are differentially expressed between SI and GH in females only. GH, group housed; SI, socially isolated.

Genes that encode miRNAs are distributed across chromosomes individually or in clusters – typically within 10 Kb of each other (Ghorai and Ghosh, 2014). Our results indicate that most of the miRNA precursors that were DE between SI and GH conditions (9 in males and 11 in females) map onto Chromosome 6 (Figure 6), some of them within clusters, which suggests that their expression might be regulated simultaneously. Consistent with this observation, most rat miRNA genes (>60) have been shown to map onto Chromosome 6 (Ghorai and Ghosh, 2014). Of note, more than 50 miRNA genes have been shown to map onto Chromosome X, but no miRNA genes have been identified on Chromosome Y in the rat genome (Ghorai and Ghosh, 2014). These results suggest that most of sex-specific effects on miRNA regulation observed in this study are independent of miRNA gene expression on sex chromosomes, as only two DE miRNAs were found on the X chromosome and none on the Y chromosome.

IsomiRs are mature miRNA isomers that vary in size by one or more nucleotides at the 3′- and/or 5′-end, with the 5′-end known as the “seed region.” Depending on where (3′- and/or 5′-end) those variations occur, the stability of the miRNA can be affected. Alternatively, 5′-isomiRs can target different mRNAs compared to the reference miRNA sequence due to shifts in the seed sequence (Tan et al., 2014). IsomiRs can be measured using small RNA sequencing and can differ between sexes (Guo et al., 2016). The current study shows a greater level of isomiR DE in the female compared to the male adBNST between SI and GH conditions (Supplementary Tables 5, 6). These results, together with the overall miRNA analysis, suggest that SI (compared to GH) in females induced more changes in isomiRs compared to the males. Differences in isomiR expression between males and females might be the product of sex differences in the actions of DICER and/or DRHOSHA on isomiRs processing.

Sequencing approaches are also used to identify novel miRNAs, which can be species specific (Huang et al., 2017). In this study, we provide evidence that SI compared to GH housing conditions change the expression of five miRNAs that were not previously identified in the female adBNST, eight in the male adBNST, and one in both male and female adBNST (Supplementary Table 4). Future studies are necessary to assess the functional role of those miRNAs.

In a first step to translate our sex-specific and stress-induced miRNA findings to humans, we compared our DE miRNA data with the 15 differentially regulated miRNAs discovered in whole blood following childhood trauma as part of the GTP (Wingo et al., 2015). Importantly, the chronic SI stressor used in the rat studies had an adolescent onset and at least part of the behavioral and biological effects described here were influenced by the developmental aspects of SI during puberty (Varty et al., 1999). Similarly, childhood trauma can have effects on stress-related psychopathology (e.g., PTSD) linked to distinct transcriptional signatures in the blood (Mehta et al., 2013). In a small, but representative subset of the same population (Wingo et al., 2015), we were able to identify blood miRNAs linked to childhood traumatic stress and describe the overlap with the SI signature in rat adBNST. Most of those overlapping miRNAs are regulated by SI in females only (*n* = 5). However, we also found overlap between male-specific SI-regulated miRNAs (*n* = 2) as well as miRNAs that are regulated by SI in both males and females (*n* = 2). The overlapping miRNAs have not been described in a previous human study of PTSD (Daskalakis et al., 2018) and need to be replicated.

It is important to note that one of the limitations of the present study is that SI is a stressor but cannot be considered a traumatic event. Thus, both SI rats and humans that experienced traumatic events experienced stress. However, the intensity of the stressor cannot be considered identical as human individuals experienced more robust stressors. As there are no published studies assessing the effects of SI stress in humans on miRNA expression, the current human study that assessed the effects of childhood trauma on miRNA expression is the most relevant published study to perform a first assessment of the translational utility of our results. This obstacle could had been partially overcome if we had assessed other behaviors in SI rats that are more relevant to traumatic events in humans such as fear conditioning or fear potentiated startle. Importantly, those behaviors include a fear component which could also affect the expression of miRNAs making the effects of SI stress on miRNA expression more difficult to interpret. Our future studies will aim to assess those behaviors on SI rats as well as on overlapping rat-human stress-regulated miRNAs identified in this study.

## Conclusion

In conclusion, the current study shows that housing conditions (either SI or GH) during adolescence regulate miRNA and isomiR expression in a sex-specific manner in the sexually dimorphic area of the adBNST, raising the possibility that the development of sex-specific pharmacotherapies to treat anxiety-like disorders is essential. Furthermore, this study suggests that the comparison of rat and human miRNA data can reveal translationally relevant information that can be leveraged for treatment and/or diagnostic development.

## Data Availability Statement

The datasets generated for this study can be found in the You may view your GSE131289 study at GEO: https://www.ncbi.nlm.nih.gov/geo/query/acc.cgi?acc=GSE131289.

## Ethics Statement

The studies involving human participants were reviewed and approved by the Institutional Review Board of Emory University School of Medicine and Grady Memorial Hospital. The patients/participants provided their written informed consent to participate in this study. The animal study was reviewed and approved by the Animal Care and Use Committee of McLean Hospital.

## Author Contributions

MM and EC designed the experiments, analyzed the results, and wrote the manuscript. MM performed the rat studies and the qRT-PCR validation. LP performed the bioinformatic analysis of the small RNA sequencing study and wrote the corresponding part of the manuscript. DP punched the adBNST tissues. MR-G and SA performed the small RNA sequencing study. ND designed and performed the bioinformatic comparison of the rat small RNA sequencing data with the existing human data, wrote the corresponding part of the manuscript, and contributed to the interpretation of those results. KR collected the human data and contributed to the interpretation of the results. EA and FS contributed to the experimental design of the validation of the small RNA sequencing results.

## Conflict of Interest

The authors declare that the research was conducted in the absence of any commercial or financial relationships that could be construed as a potential conflict of interest.

## Footnotes

1 https://bcbio-nextgen.readthedocs.org/en/latest/

2 http://www.bioinformatics.babraham.ac.uk/projects/fastqc/

3 https://github.com/lpantano/bcbioSmallRna
