# Supplementary material for: Sex-Dependent Changes in miRNA Expression in the Bed Nucleus of the Stria Terminalis Following Stress
Source: Front Mol Neurosci. 2019 Oct 4;12:236. doi: 10.3389/fnmol.2019.00236 (PMC6788329; doi:10.3389/fnmol.2019.00236)
Supplement: Supplementary file 1 [file Data_Sheet_1.docx]

**Methods**

**Estradiol measurement using ELISA**

Estradiol levels in plasma were measured using an ELISA kit (E2 ELISA, Calbiotech, Inc). The sensitivity of the kit is <3 pg/ml. Absorbance of the colorimetric reaction was read at 450 nm with a microplate reader and the unknowns were compared to a standard curve to calculate estradiol concentrations.

**
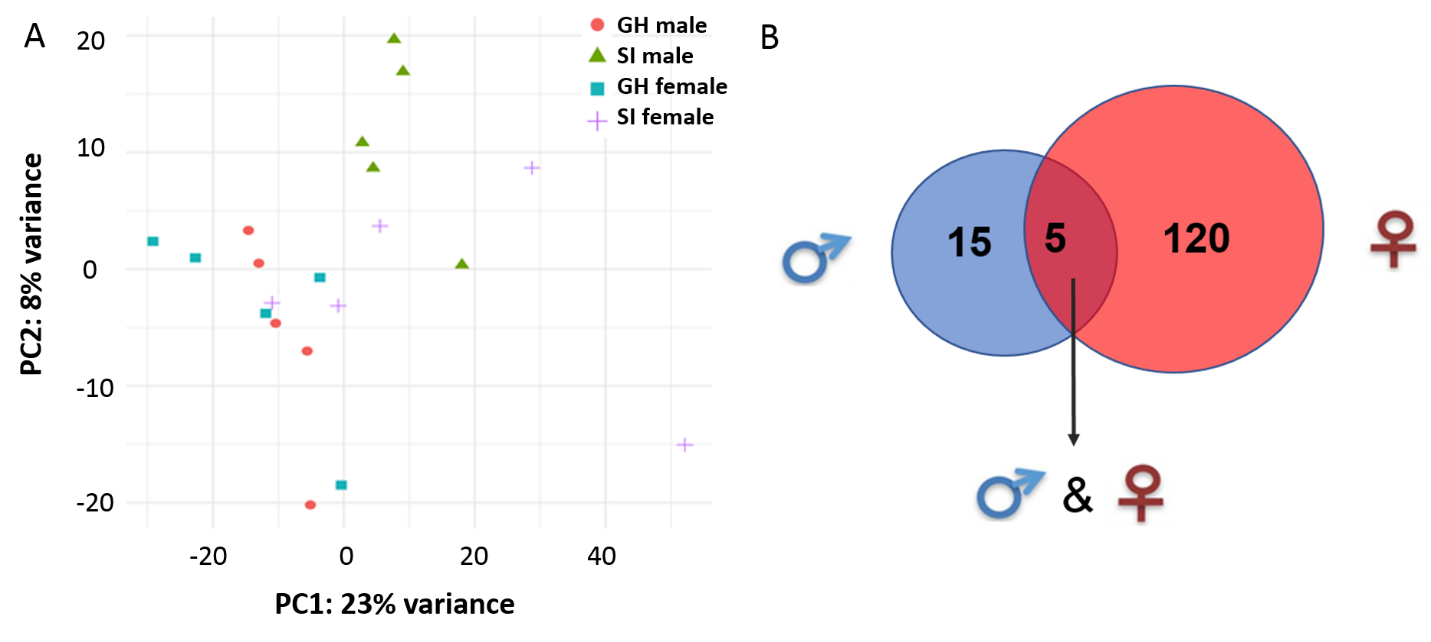
**

**Supplementary Figure 1.** Principal component analysis of isomiRs measured using small RNA sequencing and number of isomiRs that are differentially expressed between SI and GH in male and female anterodorsal bed nucleus of the stria terminalis (adBNST). **(A)** Principal component analysis showing the first two components of the regularized log2 transformed isomiR abundance data. Every dot represents a sample and they are colored by the condition information. **(B)** The Venn diagram that indicates the number of isomiRs that were significantly regulated by SI (compared to group-housed controls) in the adBNST in males only (blue), in females only (red), or in both males and females (overlap of blue and red). GH, group housed; SI, socially isolated.


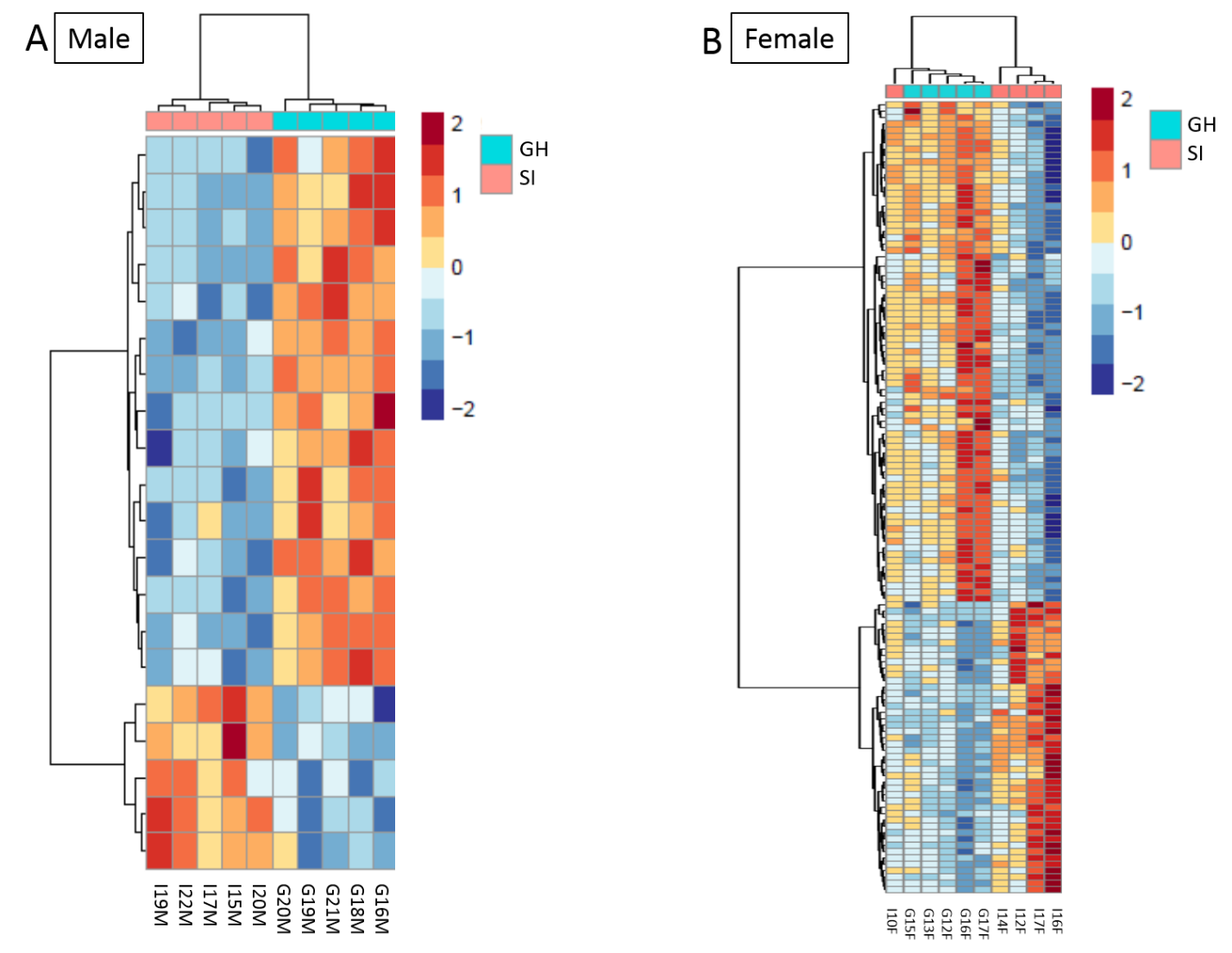


**Supplementary Figure 2.** Housing condition in adolescence induces sex-specific isomiR expression in the adBNST in adulthood. Small RNA sequencing was performed in samples of adBNST and differential expression (DE) analysis was performed to compare the expression levels between GH males vs SI males and GH females vs SI females. Color represents the isomiR z-score expression. The median of the expression is used for each group. **(A)** SI-regulated isomiRs in males and **(B)** SI-regulated isomiRs in females; n=5/group. GH, group housed; SI, socially isolated. For complete lists of DE expressed isomiRs in males and females, see **Supplementary Tables 5 and 6**.

**
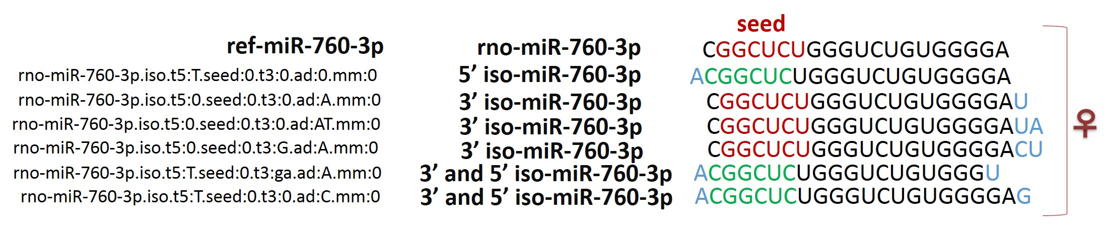
**

**Supplementary Figure 3.** Example of different isomiRs of miR-760-3p that were identified to be differentially expressed in SI compared to GH using small RNA sequencing in the female anterodorsal bed nucleus of the stria terminalis (adBNST). The first line shows the reference miR-760-3p and the lines below show different isomiRs that demonstrate additions of nucleotides in the -3p, -5p or -3p and -5p ends. Deletions of nucleotides can also be observed. Edits in the -5p end of the miRNA sequence can potentially shift the seed sequence.

**
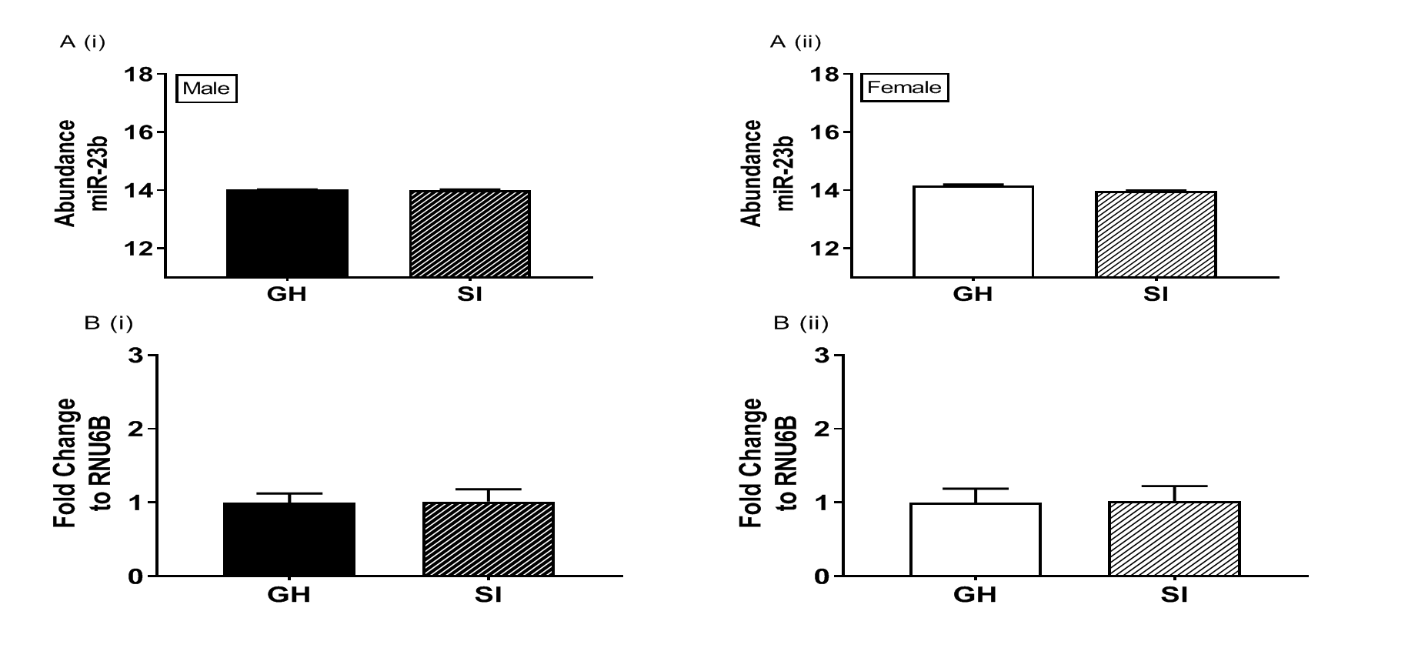
**

**Supplementary Figure 4.** MiR-23b-3p as a stable miRNA in the anterodorsal bed nucleus of the stria terminalis (adBNST). **(A)** MiR-23b-3p was identified to be stable in the adBNST using small RNA sequencing. **(B)** MiR-23b-3p and RNU6B were measured in the adBNST using qRT-PCR; miR-23b-3p expression was normalized to RNU6B to confirm that miR-23b-3p is stably expressed. GH, group housed; SI, socially isolated.

**Supplementary Figure 5.** Effects of housing condition during adolescence on estradiol levels in adulthood as measured by ELISA. GH, group housed; SI, socially isolated.

**miRNAs that are significantly regulated by SI in both males and females**

**Male expression**

| **miRNA ID** | baseMean | log2Fold  Change | lfcSE | stat | pvalue | padj |
| --- | --- | --- | --- | --- | --- | --- |
| **rno-miR-22-3p** | 8846.852 | -1.28071 | 0.25655 | -4.99206 | 5.97E-07 | 0.00023 |
| **rno-miR-341** | 722.431 | -1.16818 | 0.284211 | -4.11027 | 3.95E-05 | 0.002174 |
| **rno-miR-338-5p** | 11017.55 | 0.968016 | 0.239304 | 4.045133 | 5.23E-05 | 0.002517 |
| **rno-miR-136-3p** | 6012.404 | -1.20066 | 0.299838 | -4.00434 | 6.22E-05 | 0.00266 |
| **rno-miR-181c-5p** | 810.7187 | -1.30327 | 0.351744 | -3.70517 | 0.000211 | 0.006256 |
| **rno-miR-135b-5p** | 1555.17 | -1.13458 | 0.326921 | -3.47051 | 0.000519 | 0.0125 |
| **rno-miR-143-3p** | 60084.15 | -0.97005 | 0.284499 | -3.40966 | 0.00065 | 0.013077 |
| **rno-miR-434-5p** | 2409 | -0.86604 | 0.259343 | -3.33936 | 0.00084 | 0.015395 |
| **rno-miR-30e-5p** | 15160.15 | -0.95842 | 0.296613 | -3.23122 | 0.001233 | 0.019774 |
| **rno-miR-29a-3p** | 39082.99 | -0.91055 | 0.291877 | -3.11962 | 0.001811 | 0.02503 |
| **rno-miR-330-5p** | 3257.094 | -0.56553 | 0.19117 | -2.95827 | 0.003094 | 0.037221 |
| **rno-let-7d-5p** | 118683.1 | 0.658312 | 0.22703 | 2.899673 | 0.003736 | 0.042299 |

**Female expression**

| **miRNA ID** | baseMean | log2Fold Change | lfcSE | stat | pvalue | padj |
| --- | --- | --- | --- | --- | --- | --- |
| **rno-miR-22-3p** | 8846.852 | -1.47656 | 0.256514 | -5.75627 | 8.60E-09 | 3.13E-06 |
| **rno-miR-341** | 722.431 | -0.91583 | 0.283979 | -3.22499 | 0.00126 | 0.01433 |
| **rno-miR-338-5p** | 11017.55 | 0.695561 | 0.239302 | 2.906623 | 0.003654 | 0.027706 |
| **rno-miR-136-3p** | 6012.404 | -0.86134 | 0.29982 | -2.87284 | 0.004068 | 0.02946 |
| **rno-miR-181c-5p** | 810.7187 | -1.23056 | 0.35166 | -3.49928 | 0.000467 | 0.009553 |
| **rno-miR-135b-5p** | 1555.17 | -1.10078 | 0.326867 | -3.36767 | 0.000758 | 0.010134 |
| **rno-miR-143-3p** | 60084.15 | -0.78162 | 0.284496 | -2.74738 | 0.006007 | 0.036324 |
| **rno-miR-434-5p** | 2409 | -0.7951 | 0.25931 | -3.06623 | 0.002168 | 0.019727 |
| **rno-miR-30e-5p** | 15160.15 | -1.1092 | 0.29661 | -3.73958 | 0.000184 | 0.008387 |
| **rno-miR-29a-3p** | 39082.99 | -0.98995 | 0.291875 | -3.39167 | 0.000695 | 0.009725 |
| **rno-miR-330-5p** | 3257.094 | -0.56295 | 0.191098 | -2.94589 | 0.00322 | 0.025447 |
| **rno-let-7d-5p** | 118683.1 | 0.594875 | 0.22703 | 2.620242 | 0.008787 | 0.047737 |

**Supplementary Table 1**. List of miRNAs that were differentially regulated between social isolation (SI) and group housed (GH) conditions in both male and female anterodorsal bed nucleus of the stria terminalis (adBNST).

**SI-regulated miRNAs in males**

| id | baseMean | log2FoldChange | lfcSE | stat | pvalue | padj |
| --- | --- | --- | --- | --- | --- | --- |
| rno-miR-22-3p | 8846.9 | -1.28 | 0.26 | -4.99 | 5.97E-07 |  |
| rno-miR-25-3p | 6589.2 | 0.80 | 0.19 | 4.33 | 1.50E-05 | 0.0014 |
| rno-miR-378a-3p | 8126.9 | 0.96 | 0.22 | 4.45 | 8.49E-06 | 0.0014 |
| rno-miR-873-5p | 1224.5 | -1.19 | 0.27 | -4.38 | 1.21E-05 | 0.0014 |
| rno-miR-300-3p | 4028.0 | -1.09 | 0.26 | -4.16 | 3.25E-05 | 0.0022 |
| rno-miR-341 | 722.4 | -1.17 | 0.28 | -4.11 | 3.95E-05 | 0.0022 |
| rno-miR-34a-5p | 639.2 | 1.25 | 0.30 | 4.13 | 3.66E-05 | 0.0022 |
| rno-miR-338-5p | 11017.6 | 0.97 | 0.24 | 4.05 | 5.23E-05 | 0.0025 |
| rno-miR-136-3p | 6012.4 | -1.20 | 0.30 | -4.00 | 6.22E-05 | 0.0027 |
| rno-miR-148b-3p | 18916.3 | 1.05 | 0.27 | 3.89 | 9.88E-05 | 0.0038 |
| rno-miR-411-5p | 11719.3 | -1.21 | 0.32 | -3.85 | 0.0001 | 0.0041 |
| rno-miR-221-3p | 13972.8 | 0.94 | 0.25 | 3.80 | 0.0001 | 0.0047 |
| rno-miR-181c-5p | 810.7 | -1.30 | 0.35 | -3.71 | 0.0002 | 0.0063 |
| rno-miR-666-3p | 661.6 | -1.12 | 0.31 | -3.64 | 0.0003 | 0.0074 |
| rno-miR-127-5p | 3298.0 | -0.95 | 0.26 | -3.61 | 0.0003 | 0.0080 |
| rno-miR-135b-5p | 1555.2 | -1.13 | 0.33 | -3.47 | 0.0005 | 0.0125 |
| rno-miR-204-3p | 398.1 | 1.43 | 0.42 | 3.43 | 0.0006 | 0.0130 |
| rno-miR-30c-5p | 39487.8 | 0.55 | 0.16 | 3.43 | 0.0006 | 0.0130 |
| rno-miR-143-3p | 60084.1 | -0.97 | 0.28 | -3.41 | 0.0007 | 0.0131 |
| rno-miR-423-3p | 6952.3 | -0.70 | 0.20 | -3.40 | 0.0007 | 0.0131 |
| rno-miR-434-5p | 2409.0 | -0.87 | 0.26 | -3.34 | 0.0008 | 0.0154 |
| rno-miR-34b-3p | 3991.2 | 1.07 | 0.33 | 3.27 | 0.0011 | 0.0191 |
| rno-miR-543-5p | 77.2 | -1.39 | 0.43 | -3.25 | 0.0012 | 0.0193 |
| rno-miR-30e-5p | 15160.1 | -0.96 | 0.30 | -3.23 | 0.0012 | 0.0198 |
| rno-miR-376a-3p | 298.5 | -1.08 | 0.34 | -3.17 | 0.0015 | 0.0235 |
| rno-miR-7b | 64372.7 | 0.46 | 0.15 | 3.14 | 0.0017 | 0.0248 |
| rno-miR-204-5p | 15658.3 | 1.31 | 0.42 | 3.11 | 0.0019 | 0.0250 |
| rno-miR-29a-3p | 39083.0 | -0.91 | 0.29 | -3.12 | 0.0018 | 0.0250 |
| rno-miR-376b-3p | 2216.0 | -1.03 | 0.33 | -3.11 | 0.0019 | 0.0250 |
| rno-let-7g-5p | 178387.6 | 0.49 | 0.16 | 3.07 | 0.0022 | 0.0279 |
| rno-miR-434-3p | 91232.6 | 0.58 | 0.19 | 3.01 | 0.0026 | 0.0322 |
| rno-miR-330-5p | 3257.1 | -0.57 | 0.19 | -2.96 | 0.0031 | 0.0372 |
| rno-miR-383-5p | 3793.8 | 0.81 | 0.28 | 2.95 | 0.0032 | 0.0377 |
| rno-let-7d-5p | 118683.1 | 0.66 | 0.23 | 2.90 | 0.0037 | 0.0423 |
| rno-miR-199a-3p | 351.6 | 0.86 | 0.30 | 2.89 | 0.0039 | 0.0428 |
| rno-miR-671 | 631.3 | 0.77 | 0.27 | 2.87 | 0.0042 | 0.0445 |
| rno-miR-329-5p | 890.4 | 0.78 | 0.27 | 2.84 | 0.0045 | 0.0473 |

**Supplementary Table 2**. List of miRNAs that were that were differentially regulated between social isolation (SI) and group housed (GH) conditions in male anterodorsal bed nucleus of the stria terminalis (adBNST).

**SI-regulated miRNAs in females**

| **Id** | **baseMean** | **log2FoldChange** | **lfcSE** | **stat** | **pvalue** | **padj** |
| --- | --- | --- | --- | --- | --- | --- |
| rno-miR-22-3p | 8846.9 | -1.48 | 0.26 | -5.76 | 8.60E-09 | 3.13E-06 |
| rno-miR-153-3p | 1041.3 | -1.72 | 0.36 | -4.73 | 2.26E-06 | 0.0004 |
| rno-miR-24-3p | 12699.2 | -1.31 | 0.29 | -4.51 | 6.55E-06 | 0.0006 |
| rno-miR-664-2-5p | 2114.1 | 1.23 | 0.27 | 4.50 | 6.82E-06 | 0.0006 |
| rno-miR-486 | 10033.2 | 1.04 | 0.26 | 4.05 | 5.16E-05 | 0.0038 |
| rno-let-7c-1-3p | 108.4 | 1.42 | 0.36 | 3.94 | 8.18E-05 | 0.0050 |
| rno-miR-6331 | 10949.8 | 0.97 | 0.25 | 3.83 | 0.0001 | 0.0066 |
| rno-miR-30e-5p | 15160.1 | -1.11 | 0.30 | -3.74 | 0.0002 | 0.0084 |
| rno-miR-760-3p | 18118.9 | 1.01 | 0.28 | 3.66 | 0.0003 | 0.0093 |
| rno-miR-770-3p | 6259.4 | 0.85 | 0.23 | 3.67 | 0.0002 | 0.0093 |
| rno-let-7i-3p | 192.7 | -1.30 | 0.38 | -3.46 | 0.0005 | 0.0096 |
| rno-miR-126a-3p | 5099.5 | -1.15 | 0.32 | -3.58 | 0.0003 | 0.0096 |
| rno-miR-129-1-3p | 812.9 | -0.98 | 0.28 | -3.49 | 0.0005 | 0.0096 |
| rno-miR-129-5p | 77744.3 | 0.72 | 0.21 | 3.49 | 0.0005 | 0.0096 |
| rno-miR-181c-5p | 810.7 | -1.23 | 0.35 | -3.50 | 0.0005 | 0.0096 |
| rno-miR-21-5p | 6391.6 | -1.17 | 0.34 | -3.43 | 0.0006 | 0.0096 |
| rno-miR-27a-3p | 4586.8 | -0.82 | 0.24 | -3.43 | 0.0006 | 0.0096 |
| rno-miR-320-3p | 35929.5 | 0.87 | 0.25 | 3.54 | 0.0004 | 0.0096 |
| rno-miR-338-3p | 428.3 | -1.50 | 0.42 | -3.61 | 0.0003 | 0.0096 |
| rno-miR-346 | 9427.4 | 0.74 | 0.21 | 3.49 | 0.0005 | 0.0096 |
| rno-miR-34c-5p | 2831.3 | -1.25 | 0.35 | -3.58 | 0.0003 | 0.0096 |
| rno-miR-382-5p | 30224.1 | 0.63 | 0.18 | 3.47 | 0.0005 | 0.0096 |
| rno-miR-872-5p | 688.2 | -1.16 | 0.34 | -3.43 | 0.0006 | 0.0096 |
| rno-miR-135a-3p | 1209.6 | 0.91 | 0.27 | 3.41 | 0.0007 | 0.0097 |
| rno-miR-376b-5p | 833.1 | -1.17 | 0.34 | -3.40 | 0.0007 | 0.0097 |
| rno-miR-29a-3p | 39083.0 | -0.99 | 0.29 | -3.39 | 0.0007 | 0.0097 |
| rno-miR-135b-5p | 1555.2 | -1.10 | 0.33 | -3.37 | 0.0008 | 0.0101 |
| rno-miR-331-3p | 367.0 | -1.16 | 0.35 | -3.36 | 0.0008 | 0.0101 |
| rno-miR-132-5p | 2428.4 | -0.73 | 0.22 | -3.31 | 0.0009 | 0.0116 |
| rno-miR-106b-3p | 910.6 | 0.79 | 0.24 | 3.27 | 0.0011 | 0.0130 |
| rno-miR-140-5p | 180.8 | -1.38 | 0.42 | -3.24 | 0.0012 | 0.0140 |
| rno-miR-341 | 722.4 | -0.92 | 0.28 | -3.22 | 0.0013 | 0.0143 |
| rno-miR-16-5p | 4337.0 | -0.98 | 0.31 | -3.15 | 0.0016 | 0.0154 |
| rno-miR-1843a-3p | 1663.8 | 0.86 | 0.27 | 3.19 | 0.0014 | 0.0154 |
| rno-miR-328a-3p | 32651.0 | 0.85 | 0.27 | 3.16 | 0.0016 | 0.0154 |
| rno-miR-485-3p | 20270.2 | 0.94 | 0.30 | 3.17 | 0.0015 | 0.0154 |
| rno-miR-485-5p | 27553.3 | 0.74 | 0.23 | 3.18 | 0.0014 | 0.0154 |
| rno-miR-92a-3p | 17965.8 | 0.64 | 0.20 | 3.16 | 0.0016 | 0.0154 |
| rno-miR-92b-3p | 144437.7 | 0.85 | 0.28 | 3.10 | 0.0019 | 0.0180 |
| rno-miR-434-5p | 2409.0 | -0.80 | 0.26 | -3.07 | 0.0022 | 0.0197 |
| rno-miR-181a-5p | 45923.5 | -0.56 | 0.19 | -3.04 | 0.0024 | 0.0212 |
| rno-miR-369-5p | 1161.2 | -1.01 | 0.34 | -3.00 | 0.0027 | 0.0234 |
| rno-miR-135a-5p | 5197.5 | -0.92 | 0.31 | -2.98 | 0.0028 | 0.0241 |
| rno-miR-24-2-5p | 608.4 | -0.97 | 0.33 | -2.96 | 0.0031 | 0.0253 |
| rno-miR-433-3p | 47796.5 | 0.63 | 0.21 | 2.95 | 0.0031 | 0.0254 |
| rno-miR-125b-1-3p | 3412.1 | 0.75 | 0.26 | 2.94 | 0.0033 | 0.0254 |
| rno-miR-330-5p | 3257.1 | -0.56 | 0.19 | -2.95 | 0.0032 | 0.0254 |
| rno-miR-338-5p | 11017.6 | 0.70 | 0.24 | 2.91 | 0.0037 | 0.0277 |
| rno-miR-15a-5p | 107.5 | -1.25 | 0.43 | -2.88 | 0.0039 | 0.0291 |
| rno-miR-1188-5p | 708.5 | 0.79 | 0.28 | 2.87 | 0.0041 | 0.0295 |
| rno-miR-136-3p | 6012.4 | -0.86 | 0.30 | -2.87 | 0.0041 | 0.0295 |
| rno-miR-1224 | 2037.8 | 0.90 | 0.32 | 2.78 | 0.0054 | 0.0349 |
| rno-miR-136-5p | 530.6 | -1.16 | 0.42 | -2.77 | 0.0057 | 0.0349 |
| rno-miR-195-5p | 683.3 | -0.97 | 0.35 | -2.77 | 0.0056 | 0.0349 |
| rno-miR-3084a-3p | 173.3 | 0.88 | 0.32 | 2.78 | 0.0054 | 0.0349 |
| rno-miR-3084b-3p | 173.3 | 0.88 | 0.32 | 2.78 | 0.0054 | 0.0349 |
| rno-miR-3084d | 173.3 | 0.88 | 0.32 | 2.78 | 0.0054 | 0.0349 |
| rno-miR-31a-5p | 377.4 | -1.13 | 0.41 | -2.77 | 0.0057 | 0.0349 |
| rno-miR-877 | 773.0 | 0.77 | 0.28 | 2.78 | 0.0055 | 0.0349 |
| rno-miR-143-3p | 60084.1 | -0.78 | 0.28 | -2.75 | 0.0060 | 0.0363 |
| rno-miR-27b-3p | 23289.9 | -0.59 | 0.21 | -2.74 | 0.0061 | 0.0363 |
| rno-miR-1843b-3p | 117.5 | 1.13 | 0.41 | 2.73 | 0.0063 | 0.0370 |
| rno-miR-3099 | 15618.4 | 0.74 | 0.27 | 2.68 | 0.0073 | 0.0421 |
| rno-miR-99a-5p | 28203.1 | -0.70 | 0.26 | -2.68 | 0.0074 | 0.0421 |
| rno-miR-98-5p | 56533.4 | 0.64 | 0.24 | 2.65 | 0.0081 | 0.0451 |
| rno-miR-3068-3p | 841.0 | -0.71 | 0.27 | -2.63 | 0.0085 | 0.0469 |
| rno-let-7d-5p | 118683.1 | 0.59 | 0.23 | 2.62 | 0.0088 | 0.0477 |
| rno-miR-212-3p | 1311.4 | -0.49 | 0.19 | -2.61 | 0.0090 | 0.0484 |

**Supplementary Table 3**. List of miRNAs that were that were differentially regulated between social isolation (SI) and group housed (GH) conditions in female anterodorsal bed nucleus of the stria terminalis (adBNST).

**SI-regulated novel miRNAs in males only**

| Novel miRNA ID | Base  Mean | log2FoldChange | lfcSE | stat | pvalue | padj |
| --- | --- | --- | --- | --- | --- | --- |
| rno-10_3509-5p | 7548.2 | 0.95 | 0.23 | 4.05 | 5E-05 | 0.006 |
| rno-6_21996-3p | 291.8 | -1.54 | 0.39 | -3.92 | 9E-05 | 0.006 |
| rno-X_27201-5p | 14118.8 | 0.93 | 0.27 | 3.45 | 0.001 | 0.018 |
| rno-1_1220-3p | 391.1 | 1.65 | 0.50 | 3.32 | 0.001 | 0.019 |
| rno-4_17822-5p | 66.2 | 1.68 | 0.50 | 3.35 | 0.001 | 0.019 |
| rno-18_12068-5p | 61350.6 | -1.06 | 0.32 | -3.26 | 0.001 | 0.021 |
| rno-1_1220-5p | 15435.8 | 1.54 | 0.52 | 2.98 | 0.003 | 0.040 |
| rno-6_21904-5p | 2686.1 | -0.83 | 0.28 | -3.01 | 0.003 | 0.040 |

**SI-regulated novel miRNAs in females only**

| Novel miRNA ID | Base  Mean | log2FoldChange | lfcSE | stat | pvalue | padj |
| --- | --- | --- | --- | --- | --- | --- |
| rno-19_12349-5p | 13016.0 | -1.40 | 0.33 | -4.21 | 3E-05 | 0.004 |
| rno-6_21996-5p | 6841.1 | 0.99 | 0.27 | 3.65 | 0.000 | 0.018 |
| rno-7_23643-3p | 1201.5 | 0.89 | 0.28 | 3.22 | 0.001 | 0.036 |
| rno-9_26461-5p | 1012.5 | 0.81 | 0.25 | 3.25 | 0.001 | 0.036 |
| rno-7_23643-5p | 6854.3 | -1.05 | 0.34 | -3.07 | 0.002 | 0.050 |

**SI-regulated novel miRNAs in males and females**

| Male |  |  |  |  |  |  |
| --- | --- | --- | --- | --- | --- | --- |
| rno-19_12701-5p | 830.8 | -1.46 | 0.41 | -3.58 | 0.000 | 0.015 |
| Female |  |  |  |  |  |  |
| rno-19_12701-5p | 830.8 | -1.36 | 0.41 | -3.35 | 0.001 | 0.036 |

**Supplementary Table 4**. List of novel miRNAs that were identified in a small RNA sequencing study to be differentially regulated between social isolation (SI) and group housed (GH) conditions in the male and female anterodorsal bed nucleus of the stria terminalis (adBNST).

**SI-regulated isomiRs in males**

| Idiiiii | | baseMean | log2FoldChange | lfcSE | stat | pvalue | padj |
| --- | --- | --- | --- | --- | --- | --- | --- |
| rno-miR-423-3p.iso.t5:0.seed:0.t3:0.ad:TT.mm:0 | 354.7482 | | -1.57816 | 0.308131 | -5.12173 | 3.03E-07 | 0.003383 |
| rno-miR-101a-3p.iso.t5:0.seed:0.t3:a.ad:CT.mm:0 | 241.7961 | | -1.91254 | 0.391646 | -4.88334 | 1.04E-06 | 0.003885 |
| rno-miR-22-3p.ref.t5:0.seed:0.t3:0.ad:0.mm:0 | 7184.945 | | -1.63039 | 0.333483 | -4.88897 | 1.01E-06 | 0.003885 |
| rno-miR-136-3p.ref.t5:0.seed:0.t3:0.ad:0.mm:0 | 2115.176 | | -1.53128 | 0.344893 | -4.43986 | 9.00E-06 | 0.020115 |
| rno-miR-300-3p.ref.t5:0.seed:0.t3:0.ad:0.mm:0 | 944.1475 | | -1.2905 | 0.289531 | -4.45721 | 8.30E-06 | 0.020115 |
| rno-miR-370-3p.ref.t5:0.seed:0.t3:0.ad:0.mm:0 | 1694.547 | | -1.22699 | 0.28016 | -4.37959 | 1.19E-05 | 0.021385 |
| rno-miR-383-5p.iso.t5:0.seed:0.t3:CT.ad:0.mm:0 | 540.2611 | | 1.214584 | 0.27899 | 4.353504 | 1.34E-05 | 0.021385 |
| rno-let-7i-5p.iso.t5:0.seed:0.t3:0.ad:T.mm:0 | 6112.237 | | 0.732001 | 0.178004 | 4.11226 | 3.92E-05 | 0.025751 |
| rno-miR-101a-3p.iso.t5:G.seed:0.t3:aa.ad:0.mm:0 | 937.08 | | -1.71246 | 0.416116 | -4.11535 | 3.87E-05 | 0.025751 |
| rno-miR-136-3p.ref.t5:c.seed:0.t3:0.ad:0.mm:0 | 2232.66 | | -1.45064 | 0.347981 | -4.16873 | 3.06E-05 | 0.025751 |
| rno-miR-148b-3p.ref.t5:0.seed:0.t3:0.ad:0.mm:0 | 8985.699 | | 1.151454 | 0.273259 | 4.213774 | 2.51E-05 | 0.025751 |
| rno-miR-221-3p.iso.t5:0.seed:0.t3:c.ad:T.mm:0 | 716.5957 | | 1.156205 | 0.277817 | 4.161743 | 3.16E-05 | 0.025751 |
| rno-miR-411-5p.ref.t5:0.seed:0.t3:0.ad:0.mm:0 | 5384.32 | | -1.42245 | 0.345073 | -4.12218 | 3.75E-05 | 0.025751 |
| rno-miR-423-3p.ref.t5:0.seed:0.t3:0.ad:0.mm:0 | 3719.737 | | -0.95573 | 0.227881 | -4.194 | 2.74E-05 | 0.025751 |
| rno-miR-434-3p.ref.t5:0.seed:0.t3:t.ad:0.mm:0 | 10415.66 | | 0.92957 | 0.217129 | 4.281188 | 1.86E-05 | 0.025751 |
| rno-miR-708-3p.ref.t5:0.seed:0.t3:0.ad:0.mm:0 | 327.0662 | | -1.17289 | 0.282247 | -4.15552 | 3.25E-05 | 0.025751 |
| rno-miR-873-5p.iso.t5:T.seed:0.t3:cct.ad:0.mm:0 | 349.1707 | | -1.43037 | 0.338677 | -4.22341 | 2.41E-05 | 0.025751 |
| rno-miR-135b-5p.ref.t5:0.seed:0.t3:0.ad:0.mm:0 | 1176.405 | | -1.58306 | 0.391571 | -4.04286 | 5.28E-05 | 0.032777 |
| rno-miR-300-3p.ref.t5:0.seed:0.t3:tc.ad:0.mm:0 | 1676.684 | | -1.24559 | 0.310803 | -4.00765 | 6.13E-05 | 0.036063 |
| rno-miR-676.ref.t5:0.seed:0.t3:0.ad:0.mm:0 | 510.3557 | | -1.1982 | 0.304672 | -3.93274 | 8.40E-05 | 0.046918 |

**Supplementary Table 5**. List of isomiRs that were differentially regulated between social isolation (SI) and group housed (GH) conditions in male anterodorsal bed nucleus of the stria terminalis (adBNST). If the name of the isomiR includes the term “iso” that means that this is an isomiR while the term “ref” refers to the reference miRNA sequence. “t5” indicates the 5p end and “t3” indicates the 3p end of the miRNA sequence; if “t5” or “t3” it’s followed by the 0 that means that there are no edits in that sequence. If additional nucleotides are presented with upper cases (ie T), this indicates additions. If additional nucleotides are presented with lower cases (ie t), this indicates deletion. “Seed” refers to changes in the nts in the seed region and is followed by 0 that indicated no changes in the seed sequence as our study did not identify any miRNAs that had changes in the seed region. Finally, the code “mm” corresponds to a mismatch in nts and if 0 means that there were no mismatches.

**SI-regulated isomiRs in females**

| Id | baseMean | log2FoldChange | lfcSE | stat | pvalue | padj |
| --- | --- | --- | --- | --- | --- | --- |
| rno-miR-22-3p.ref.t5:0.seed:0.t3:0.ad:0.mm:0 | 7184.945 | -1.89991 | 0.333431 | -5.69806 | 1.21E-08 | 2.42E-05 |
| rno-miR-129-5p.ref.t5:0.seed:0.t3:c.ad:0.mm:0 | 10619.46 | 1.035074 | 0.214119 | 4.834104 | 1.34E-06 | 0.001333 |
| rno-miR-129-5p.iso.t5:0.seed:0.t3:c.ad:T.mm:0 | 2579.923 | 0.923371 | 0.19957 | 4.626794 | 3.71E-06 | 0.001664 |
| rno-miR-153-3p.ref.t5:0.seed:0.t3:0.ad:0.mm:0 | 569.2887 | -2.05136 | 0.441529 | -4.64604 | 3.38E-06 | 0.001664 |
| rno-miR-181a-5p.ref.t5:0.seed:0.t3:0.ad:0.mm:0 | 11267.97 | -1.21959 | 0.269011 | -4.53362 | 5.80E-06 | 0.001664 |
| rno-miR-24-3p.ref.t5:0.seed:0.t3:0.ad:0.mm:0 | 5912.673 | -1.64925 | 0.360971 | -4.56893 | 4.90E-06 | 0.001664 |
| rno-miR-760-3p.iso.t5:0.seed:0.t3:0.ad:A.mm:0 | 294.6333 | 1.550108 | 0.342032 | 4.532051 | 5.84E-06 | 0.001664 |
| rno-miR-24-3p.iso.t5:0.seed:0.t3:0.ad:T.mm:0 | 1141.357 | -1.66454 | 0.3964 | -4.19915 | 2.68E-05 | 0.00598 |
| rno-miR-664-2-5p.iso.t5:0.seed:0.t3:A.ad:0.mm:0 | 1038.753 | 1.131637 | 0.269599 | 4.197489 | 2.70E-05 | 0.00598 |
| rno-miR-24-3p.ref.t5:0.seed:0.t3:g.ad:0.mm:0 | 2490.117 | -1.51103 | 0.362353 | -4.17005 | 3.05E-05 | 0.006072 |
| rno-miR-126a-3p.ref.t5:0.seed:0.t3:0.ad:0.mm:0 | 3178.244 | -1.47754 | 0.366758 | -4.02866 | 5.61E-05 | 0.008444 |
| rno-miR-181a-5p.iso.t5:0.seed:0.t3:T.ad:0.mm:0 | 1112.005 | -1.27769 | 0.317883 | -4.01935 | 5.84E-05 | 0.008444 |
| rno-miR-370-3p.ref.t5:0.seed:0.t3:0.ad:0.mm:0 | 1694.547 | -1.12885 | 0.280051 | -4.03089 | 5.56E-05 | 0.008444 |
| rno-miR-770-3p.iso.t5:0.seed:0.t3:CT.ad:0.mm:0 | 217.2874 | 1.187794 | 0.295793 | 4.01563 | 5.93E-05 | 0.008444 |
| rno-let-7d-5p.iso.t5:0.seed:0.t3:tt.ad:A.mm:0 | 337.3739 | 1.231881 | 0.31614 | 3.89663 | 9.75E-05 | 0.008465 |
| rno-miR-124-3p.iso.t5:0.seed:0.t3:A.ad:0.mm:0 | 2064.574 | -1.40273 | 0.360085 | -3.89555 | 9.80E-05 | 0.008465 |
| rno-miR-124-3p.iso.t5:T.seed:0.t3:A.ad:0.mm:0 | 664.0759 | -1.48064 | 0.373704 | -3.96208 | 7.43E-05 | 0.008465 |
| rno-miR-181a-5p.ref.t5:0.seed:0.t3:t.ad:0.mm:0 | 7317.931 | -0.95515 | 0.244251 | -3.91054 | 9.21E-05 | 0.008465 |
| rno-miR-24-3p.ref.t5:0.seed:0.t3:ag.ad:0.mm:0 | 2473.764 | -1.2625 | 0.32488 | -3.88605 | 0.000102 | 0.008465 |
| rno-miR-29a-3p.ref.t5:0.seed:0.t3:a.ad:0.mm:0 | 14254.58 | -1.42189 | 0.358764 | -3.96329 | 7.39E-05 | 0.008465 |
| rno-miR-30e-5p.iso.t5:0.seed:0.t3:CT.ad:0.mm:0 | 11392.42 | -1.26341 | 0.324643 | -3.8917 | 9.95E-05 | 0.008465 |
| rno-miR-486.iso.t5:0.seed:0.t3:0.ad:A.mm:0 | 1996.124 | 0.970591 | 0.248113 | 3.911886 | 9.16E-05 | 0.008465 |
| rno-miR-6331.ref.t5:0.seed:0.t3:gc.ad:0.mm:0 | 2132.304 | 0.840414 | 0.213752 | 3.931719 | 8.43E-05 | 0.008465 |
| rno-miR-664-2-5p.ref.t5:0.seed:0.t3:0.ad:0.mm:0 | 434.3535 | 1.355557 | 0.345251 | 3.926298 | 8.63E-05 | 0.008465 |
| rno-miR-27a-3p.ref.t5:0.seed:0.t3:0.ad:0.mm:0 | 274.9969 | -1.87442 | 0.492747 | -3.80401 | 0.000142 | 0.011356 |
| rno-let-7i-3p.ref.t5:0.seed:0.t3:0.ad:0.mm:0 | 172.5737 | -1.62663 | 0.430818 | -3.77568 | 0.00016 | 0.011752 |
| rno-miR-136-5p.ref.t5:0.seed:0.t3:a.ad:0.mm:0 | 154.0286 | -2.14671 | 0.569827 | -3.7673 | 0.000165 | 0.011752 |
| rno-miR-23a-3p.iso.t5:0.seed:0.t3:A.ad:0.mm:0 | 5967.707 | -0.83786 | 0.222118 | -3.77212 | 0.000162 | 0.011752 |
| rno-miR-486.ref.t5:0.seed:0.t3:0.ad:0.mm:0 | 2855.608 | 0.884675 | 0.236059 | 3.74769 | 0.000178 | 0.012271 |
| rno-miR-181a-5p.iso.t5:0.seed:0.t3:TT.ad:0.mm:0 | 538.5899 | -1.22846 | 0.335037 | -3.66665 | 0.000246 | 0.015313 |
| rno-miR-29a-3p.ref.t5:0.seed:0.t3:0.ad:0.mm:0 | 8840.746 | -1.31263 | 0.357536 | -3.67132 | 0.000241 | 0.015313 |
| rno-miR-320-3p.ref.t5:0.seed:0.t3:a.ad:0.mm:0 | 1160.461 | 0.904622 | 0.245892 | 3.678944 | 0.000234 | 0.015313 |
| rno-miR-376b-5p.ref.t5:0.seed:0.t3:a.ad:0.mm:0 | 572.0873 | -1.41007 | 0.386723 | -3.6462 | 0.000266 | 0.016082 |
| rno-miR-125b-5p.iso.t5:0.seed:0.t3:ga.ad:A.mm:0 | 334.3103 | -0.89623 | 0.246916 | -3.62971 | 0.000284 | 0.016599 |
| rno-miR-16-5p.ref.t5:0.seed:0.t3:0.ad:0.mm:0 | 2768.239 | -1.36527 | 0.377609 | -3.61558 | 0.0003 | 0.016599 |
| rno-miR-191a-5p.iso.t5:0.seed:0.t3:g.ad:T.mm:0 | 763.441 | -0.8627 | 0.238238 | -3.62118 | 0.000293 | 0.016599 |
| rno-miR-129-5p.ref.t5:0.seed:0.t3:gc.ad:0.mm:0 | 2187.926 | 0.775822 | 0.219316 | 3.537467 | 0.000404 | 0.018542 |
| rno-miR-135b-5p.ref.t5:0.seed:0.t3:0.ad:0.mm:0 | 1176.405 | -1.38479 | 0.391334 | -3.53864 | 0.000402 | 0.018542 |
| rno-miR-153-3p.iso.t5:0.seed:0.t3:AT.ad:0.mm:0 | 150.7487 | -1.78487 | 0.500926 | -3.56315 | 0.000366 | 0.018542 |
| rno-miR-27a-3p.iso.t5:0.seed:0.t3:cgc.ad:T.mm:0 | 3076.396 | -0.94392 | 0.265771 | -3.55161 | 0.000383 | 0.018542 |
| rno-miR-27b-3p.iso.t5:0.seed:0.t3:0.ad:T.mm:0 | 253.275 | -1.35873 | 0.384462 | -3.53411 | 0.000409 | 0.018542 |
| rno-miR-27b-3p.ref.t5:0.seed:0.t3:gc.ad:0.mm:0 | 3076.396 | -0.94392 | 0.265771 | -3.55161 | 0.000383 | 0.018542 |
| rno-miR-30e-5p.iso.t5:t.seed:0.t3:CT.ad:0.mm:0 | 372.5035 | -1.36006 | 0.381191 | -3.56792 | 0.00036 | 0.018542 |
| rno-miR-6331.iso.t5:0.seed:0.t3:c.ad:T.mm:0 | 335.6223 | 1.17729 | 0.329444 | 3.573569 | 0.000352 | 0.018542 |
| rno-miR-29a-3p.iso.t5:0.seed:0.t3:a.ad:T.mm:0 | 328.3512 | -1.46756 | 0.416115 | -3.5268 | 0.000421 | 0.018638 |
| rno-miR-760-3p.iso.t5:T.seed:0.t3:0.ad:0.mm:0 | 1628.625 | 1.062564 | 0.303347 | 3.502802 | 0.00046 | 0.019957 |
| rno-miR-27a-3p.ref.t5:0.seed:0.t3:gc.ad:0.mm:0 | 185.2496 | -1.35417 | 0.38935 | -3.47801 | 0.000505 | 0.020985 |
| rno-miR-27b-3p.iso.t5:0.seed:0.t3:tgc.ad:C.mm:0 | 185.2496 | -1.35417 | 0.38935 | -3.47801 | 0.000505 | 0.020985 |
| rno-miR-129-1-3p.iso.t5:0.seed:0.t3:0.ad:C.mm:0 | 506.6939 | -1.13016 | 0.327316 | -3.4528 | 0.000555 | 0.021274 |
| rno-miR-129-2-3p.ref.t5:0.seed:0.t3:at.ad:0.mm:0 | 506.6939 | -1.13016 | 0.327316 | -3.4528 | 0.000555 | 0.021274 |
| rno-miR-136-3p.ref.t5:c.seed:0.t3:0.ad:0.mm:0 | 2232.66 | -1.20302 | 0.347931 | -3.45765 | 0.000545 | 0.021274 |
| rno-miR-6331.ref.t5:c.seed:0.t3:gc.ad:0.mm:0 | 2264.241 | 0.820579 | 0.236748 | 3.466052 | 0.000528 | 0.021274 |
| rno-miR-760-3p.iso.t5:0.seed:0.t3:0.ad:AT.mm:0 | 178.4185 | 1.391987 | 0.404261 | 3.443283 | 0.000575 | 0.021622 |
| rno-miR-135a-5p.ref.t5:0.seed:0.t3:0.ad:0.mm:0 | 4551.581 | -1.22877 | 0.360939 | -3.40436 | 0.000663 | 0.024249 |
| rno-miR-24-2-5p.ref.t5:0.seed:0.t3:t.ad:0.mm:0 | 322.1556 | -1.27025 | 0.37367 | -3.39939 | 0.000675 | 0.024249 |
| rno-miR-3099.iso.t5:0.seed:0.t3:ga.ad:A.mm:0 | 372.9153 | 1.233365 | 0.363063 | 3.397108 | 0.000681 | 0.024249 |
| rno-miR-6331.iso.t5:c.seed:0.t3:c.ad:T.mm:0 | 637.2202 | 1.044094 | 0.308176 | 3.387976 | 0.000704 | 0.024631 |
| rno-miR-92b-3p.ref.t5:t.seed:0.t3:0.ad:0.mm:0 | 828.7248 | 1.00928 | 0.298946 | 3.37613 | 0.000735 | 0.025273 |
| rno-miR-29a-3p.iso.t5:0.seed:0.t3:T.ad:0.mm:0 | 715.3708 | -1.22198 | 0.363211 | -3.36439 | 0.000767 | 0.025615 |
| rno-miR-30c-5p.ref.t5:0.seed:0.t3:c.ad:0.mm:0 | 455.0281 | -1.27852 | 0.381189 | -3.35403 | 0.000796 | 0.025615 |
| rno-miR-320-3p.ref.t5:0.seed:0.t3:0.ad:0.mm:0 | 9470.533 | 0.711783 | 0.212099 | 3.355898 | 0.000791 | 0.025615 |
| rno-miR-92a-3p.iso.t5:0.seed:0.t3:T.ad:A.mm:0 | 1932.036 | 0.73554 | 0.218997 | 3.358668 | 0.000783 | 0.025615 |
| rno-miR-181d-5p.ref.t5:0.seed:0.t3:0.ad:0.mm:0 | 577.8691 | -0.9169 | 0.273743 | -3.34949 | 0.00081 | 0.025624 |
| rno-miR-92a-3p.iso.t5:0.seed:0.t3:T.ad:AA.mm:0 | 744.6618 | 0.926659 | 0.277387 | 3.340677 | 0.000836 | 0.026039 |
| rno-miR-129-1-3p.iso.t5:0.seed:0.t3:TAT.ad:0.mm:0 | 147.1977 | -1.69352 | 0.511955 | -3.30795 | 0.00094 | 0.026976 |
| rno-miR-129-5p.iso.t5:0.seed:0.t3:gc.ad:T.mm:0 | 409.1818 | 1.113824 | 0.336259 | 3.312397 | 0.000925 | 0.026976 |
| rno-miR-21-5p.iso.t5:0.seed:0.t3:C.ad:A.mm:0 | 523.0474 | -1.29658 | 0.389929 | -3.32516 | 0.000884 | 0.026976 |
| rno-miR-22-3p.ref.t5:0.seed:0.t3:gt.ad:0.mm:0 | 1081.793 | -0.81443 | 0.246808 | -3.29984 | 0.000967 | 0.026976 |
| rno-miR-29a-3p.ref.t5:0.seed:0.t3:ta.ad:0.mm:0 | 2981.9 | -1.27483 | 0.385733 | -3.30495 | 0.00095 | 0.026976 |
| rno-miR-34c-5p.ref.t5:0.seed:0.t3:0.ad:0.mm:0 | 2527.221 | -1.51594 | 0.460744 | -3.29021 | 0.001001 | 0.026976 |
| rno-miR-485-3p.ref.t5:0.seed:0.t3:0.ad:0.mm:0 | 11118.56 | 0.96472 | 0.292612 | 3.296922 | 0.000978 | 0.026976 |
| rno-miR-485-5p.ref.t5:0.seed:0.t3:c.ad:0.mm:0 | 2097.847 | 0.893878 | 0.271363 | 3.294035 | 0.000988 | 0.026976 |
| rno-miR-760-3p.ref.t5:0.seed:0.t3:0.ad:0.mm:0 | 1279.279 | 1.025919 | 0.310837 | 3.300504 | 0.000965 | 0.026976 |
| rno-miR-770-3p.iso.t5:0.seed:0.t3:CTG.ad:0.mm:0 | 909.1296 | 0.876308 | 0.263953 | 3.319943 | 0.0009 | 0.026976 |
| rno-miR-186-5p.iso.t5:0.seed:0.t3:T.ad:0.mm:0 | 1213.113 | -1.19085 | 0.36371 | -3.27418 | 0.00106 | 0.027837 |
| rno-miR-341.ref.t5:0.seed:0.t3:0.ad:0.mm:0 | 226.5294 | -1.43426 | 0.438098 | -3.27383 | 0.001061 | 0.027837 |
| rno-miR-132-5p.ref.t5:0.seed:0.t3:0.ad:0.mm:0 | 1179.281 | -1.03769 | 0.318119 | -3.26197 | 0.001106 | 0.028634 |
| rno-miR-181b-5p.ref.t5:0.seed:0.t3:0.ad:0.mm:0 | 3853.558 | -0.75815 | 0.232668 | -3.25849 | 0.00112 | 0.028634 |
| rno-miR-29a-3p.iso.t5:0.seed:0.t3:0.ad:A.mm:0 | 2369.282 | -1.20022 | 0.368801 | -3.25439 | 0.001136 | 0.028682 |
| rno-miR-181c-5p.ref.t5:0.seed:0.t3:0.ad:0.mm:0 | 301.9769 | -1.42932 | 0.439794 | -3.24997 | 0.001154 | 0.028767 |
| rno-miR-21-5p.ref.t5:0.seed:0.t3:0.ad:0.mm:0 | 2866.493 | -1.44145 | 0.44453 | -3.24265 | 0.001184 | 0.028965 |
| rno-miR-30a-5p.ref.t5:0.seed:0.t3:0.ad:0.mm:0 | 3815.524 | -1.03516 | 0.319395 | -3.241 | 0.001191 | 0.028965 |
| rno-miR-30e-5p.ref.t5:0.seed:0.t3:aag.ad:0.mm:0 | 461.3073 | -1.31948 | 0.412406 | -3.19947 | 0.001377 | 0.033076 |
| rno-miR-21-5p.iso.t5:0.seed:0.t3:C.ad:0.mm:0 | 2076.793 | -1.45963 | 0.458504 | -3.18347 | 0.001455 | 0.033651 |
| rno-miR-30a-5p.ref.t5:0.seed:0.t3:ag.ad:0.mm:0 | 569.7037 | -1.2497 | 0.392877 | -3.18089 | 0.001468 | 0.033651 |
| rno-miR-369-5p.ref.t5:0.seed:0.t3:c.ad:0.mm:0 | 528.5336 | -1.31107 | 0.411362 | -3.18713 | 0.001437 | 0.033651 |
| rno-miR-760-3p.iso.t5:0.seed:0.t3:G.ad:A.mm:0 | 563.2995 | 0.919511 | 0.28849 | 3.187324 | 0.001436 | 0.033651 |
| rno-miR-27b-3p.ref.t5:0.seed:0.t3:0.ad:0.mm:0 | 10907.91 | -0.783 | 0.247772 | -3.16014 | 0.001577 | 0.035731 |
| rno-miR-125b-5p.iso.t5:0.seed:0.t3:a.ad:T.mm:0 | 2039.533 | -0.63961 | 0.203396 | -3.14466 | 0.001663 | 0.03684 |
| rno-miR-92b-3p.iso.t5:0.seed:0.t3:0.ad:ACT.mm:0 | 101.4273 | 1.58508 | 0.503796 | 3.146273 | 0.001654 | 0.03684 |
| rno-miR-143-3p.ref.t5:0.seed:0.t3:a.ad:0.mm:0 | 14307.74 | -1.04205 | 0.33214 | -3.1374 | 0.001705 | 0.036853 |
| rno-miR-338-3p.ref.t5:0.seed:0.t3:ga.ad:0.mm:0 | 260.8741 | -1.59953 | 0.510226 | -3.13495 | 0.001719 | 0.036853 |
| rno-miR-92b-3p.iso.t5:0.seed:0.t3:c.ad:T.mm:0 | 4316.879 | 0.838403 | 0.26693 | 3.140911 | 0.001684 | 0.036853 |
| rno-miR-204-5p.ref.t5:0.seed:0.t3:t.ad:0.mm:0 | 354.4133 | -1.47216 | 0.470194 | -3.13096 | 0.001742 | 0.036961 |
| rno-miR-1843a-3p.ref.t5:0.seed:0.t3:0.ad:0.mm:0 | 745.2142 | 0.885286 | 0.283168 | 3.12636 | 0.00177 | 0.037148 |
| rno-miR-124-3p.ref.t5:0.seed:0.t3:0.ad:0.mm:0 | 3564.513 | -1.05231 | 0.33809 | -3.1125 | 0.001855 | 0.038532 |
| rno-miR-186-5p.ref.t5:0.seed:0.t3:t.ad:0.mm:0 | 623.5615 | -0.95751 | 0.308472 | -3.10403 | 0.001909 | 0.039243 |
| rno-miR-129-5p.iso.t5:0.seed:0.t3:c.ad:AA.mm:0 | 370.7575 | 0.9712 | 0.314095 | 3.092057 | 0.001988 | 0.039375 |
| rno-miR-330-5p.iso.t5:0.seed:0.t3:T.ad:0.mm:0 | 363.6987 | -1.47011 | 0.474658 | -3.09719 | 0.001954 | 0.039375 |
| rno-miR-770-3p.iso.t5:0.seed:0.t3:CTG.ad:T.mm:0 | 466.846 | 0.905658 | 0.29248 | 3.096477 | 0.001958 | 0.039375 |
| rno-miR-99a-5p.ref.t5:0.seed:0.t3:0.ad:0.mm:0 | 11450.22 | -1.0125 | 0.327556 | -3.09107 | 0.001994 | 0.039375 |
| rno-miR-6331.ref.t5:0.seed:0.t3:c.ad:0.mm:0 | 840.3727 | 0.888481 | 0.288561 | 3.079001 | 0.002077 | 0.040603 |
| rno-miR-30a-5p.iso.t5:0.seed:0.t3:0.ad:T.mm:0 | 306.1342 | -1.15211 | 0.37467 | -3.07499 | 0.002105 | 0.040753 |
| rno-miR-99a-5p.ref.t5:0.seed:0.t3:g.ad:0.mm:0 | 12693.62 | -0.88707 | 0.28881 | -3.07146 | 0.00213 | 0.040841 |
| rno-miR-29a-3p.iso.t5:C.seed:0.t3:a.ad:0.mm:0 | 4166.941 | -1.09677 | 0.35742 | -3.06856 | 0.002151 | 0.040847 |
| rno-miR-92b-3p.ref.t5:0.seed:0.t3:c.ad:0.mm:0 | 6092.687 | 0.724185 | 0.237235 | 3.052611 | 0.002269 | 0.042675 |
| rno-miR-92b-3p.iso.t5:0.seed:0.t3:0.ad:TTT.mm:0 | 1427.301 | 0.96504 | 0.317198 | 3.042393 | 0.002347 | 0.043739 |
| rno-miR-320-3p.iso.t5:0.seed:0.t3:a.ad:T.mm:0 | 841.5301 | 0.893457 | 0.294037 | 3.038589 | 0.002377 | 0.043884 |
| rno-miR-320-3p.iso.t5:0.seed:0.t3:A.ad:0.mm:0 | 4555.818 | 0.635514 | 0.209532 | 3.033022 | 0.002421 | 0.043889 |
| rno-miR-760-3p.iso.t5:T.seed:0.t3:ga.ad:A.mm:0 | 236.6757 | 0.985127 | 0.324525 | 3.035599 | 0.002401 | 0.043889 |
| rno-miR-328a-3p.iso.t5:0.seed:0.t3:t.ad:A.mm:0 | 2580.534 | 0.785208 | 0.259856 | 3.021701 | 0.002514 | 0.045154 |
| rno-miR-23b-3p.iso.t5:0.seed:0.t3:AC.ad:T.mm:0 | 4346.014 | -0.54048 | 0.179535 | -3.01045 | 0.002609 | 0.046443 |
| rno-miR-103-3p.iso.t5:0.seed:0.t3:a.ad:T.mm:0 | 1726.709 | -0.74672 | 0.250027 | -2.98657 | 0.002821 | 0.047098 |
| rno-miR-23b-3p.iso.t5:0.seed:0.t3:A.ad:0.mm:0 | 4107.775 | -0.64934 | 0.216361 | -3.00121 | 0.002689 | 0.047098 |
| rno-miR-29c-3p.ref.t5:0.seed:0.t3:a.ad:0.mm:0 | 476.0346 | -1.61988 | 0.541514 | -2.99138 | 0.002777 | 0.047098 |
| rno-miR-411-5p.ref.t5:0.seed:0.t3:0.ad:0.mm:0 | 5384.32 | -1.03198 | 0.345007 | -2.99118 | 0.002779 | 0.047098 |
| rno-miR-485-5p.ref.t5:0.seed:0.t3:0.ad:0.mm:0 | 4502.675 | 0.653143 | 0.218176 | 2.993653 | 0.002757 | 0.047098 |
| rno-miR-486.iso.t5:0.seed:0.t3:0.ad:T.mm:0 | 1982.098 | 0.859975 | 0.288073 | 2.985267 | 0.002833 | 0.047098 |
| rno-miR-6331.iso.t5:c.seed:0.t3:c.ad:A.mm:0 | 1210.142 | 0.834415 | 0.279522 | 2.985153 | 0.002834 | 0.047098 |
| rno-miR-760-3p.iso.t5:T.seed:0.t3:0.ad:C.mm:0 | 167.3271 | 1.209008 | 0.40485 | 2.986314 | 0.002824 | 0.047098 |
| rno-miR-143-3p.iso.t5:0.seed:0.t3:a.ad:T.mm:0 | 31660.2 | -0.96347 | 0.32519 | -2.96279 | 0.003049 | 0.048632 |
| rno-miR-204-5p.iso.t5:0.seed:0.t3:0.ad:T.mm:0 | 96.47521 | -1.68455 | 0.567681 | -2.96742 | 0.003003 | 0.048632 |
| rno-miR-380-3p.ref.t5:0.seed:0.t3:0.ad:0.mm:0 | 1936.305 | -0.98274 | 0.331283 | -2.96645 | 0.003013 | 0.048632 |
| rno-miR-382-5p.iso.t5:ga.seed:0.t3:CT.ad:0.mm:0 | 466.2792 | 0.792899 | 0.267462 | 2.96453 | 0.003031 | 0.048632 |
| rno-miR-92b-3p.ref.t5:0.seed:0.t3:0.ad:0.mm:0 | 67765.06 | 0.716429 | 0.241271 | 2.969401 | 0.002984 | 0.048632 |

**Supplementary Table 6**. List of isomiRs that were differentially regulated between social isolation (SI) and group housed (GH) conditions in female anterodorsal bed nucleus of stria terminalis (adBNST). If the name of the isomiR includes the term “iso” that means that this is an isomiR while the term “ref” refers to the reference miRNA sequence. “t5” indicates the 5p end and “t3” indicates the 3p end of the miRNA sequence; if “t5” or “t3” it’s followed by the 0 that means that there are no edits in that sequence. If additional nucleotides are presented with upper cases (ie T), this indicates additions. If additional nucleotides are presented with lower cases (ie t), this indicates deletion. “Seed” refers to changes in the nts in the seed region and is followed by 0 that indicated no changes in the seed sequence as our study did not identify any miRNAs that had changes in the seed region. Finally, the code “mm” corresponds to a mismatch in nts and if 0 means that there were no mismatches.

| **Characteristics** |  |
| --- | --- |
| **N=21**  **Age** | 44.05±2.91 |
| **Sex (% female)** | 66.66% |
| **Race (% of African American)** | 100% |
| **% PTSD** | 52.4% |
| **% Depression** | 57.1% |
|  |  |

**Supplementary Table 7**. Characteristics of the human participants in the Grady Trauma Project (GTP) sample analyzed in this study.
